# BAMBI: A new method for automated assessment of bidirectional early-life interaction between maternal behavior and pup vocalization in mouse dam-pup dyads

**DOI:** 10.3389/fnbeh.2023.1139254

**Published:** 2023-03-03

**Authors:** Carmen Winters, Wim Gorssen, Markus Wöhr, Rudi D’Hooge

**Affiliations:** ^1^Laboratory of Biological Psychology, KU Leuven, Leuven, Belgium; ^2^Leuven Brain Institute, KU Leuven, Leuven, Belgium; ^3^Department of Biosystems, Center for Animal Breeding and Genetics, KU Leuven, Leuven, Belgium; ^4^Social and Affective Neuroscience Research Group, Laboratory of Biological Psychology, Research Unit Brain and Cognition, Faculty of Psychology and Educational Sciences, KU Leuven, Leuven, Belgium; ^5^Behavioral Neuroscience, Experimental and Biological Psychology, Faculty of Psychology, Philipps-University Marburg, Marburg, Germany; ^6^Center for Mind, Brain and Behavior, Philipps-University Marburg, Marburg, Germany

**Keywords:** early-life, communication, mouse, behavior, pup retrieval, ultrasonic vocalizations, automation

## Abstract

Vital early-life dyadic interaction in mice requires a pup to signal its needs adequately, and a dam to recognize and respond to the pup’s cues accurately and timely. Previous research might have missed important biological and/or environmental elements of this complex bidirectional interaction, because it often focused on one dyadic member only. In laboratory rodents, the Pup Retrieval Test (PRT) is the leading procedure to assess pup-directed maternal care. The present study describes BAMBI (Bidirectional Automated Mother-pup Behavioral Interaction test), a novel automated PRT methodology based on synchronous video recording of maternal behavior and audio recording of pup vocalizations, which allows to assess bidirectional dam-pup dyadic interaction. We were able to estimate pup retrieval and pup vocalization parameters accurately in 156 pups from 29 dams on postnatal days (PND) 5, 7, 9, 11, and 13. Moreover, we showed an association between number of emitted USVs and retrieval success, indicating dyadic interdependency and bidirectionality. BAMBI is a promising new automated home-cage behavioral method that can be applied to both basic and preclinical studies investigating complex phenotypes related to early-life social development.

## Introduction

Neonatal mouse pups depend on their dam for nutrition, thermoregulation, and protection (Nowak et al., 2000). They produce acoustic signals to communicate their vital needs, and particularly ultrasonic vocalizations (USVs) are essential to evoke maternal care behaviors, such as retrieval in pups that have dangerously strayed from the nest (Wöhr et al., 2010; Bornstein et al., 2017). Early-life USVs can be used to study the genetic and neural basis of early-life communication and to assess early-life communicative defects and their impact on social development (Hahn and Lavooy, 2005; Scattoni et al., 2009; Reynolds et al., 2017; Potasiewicz et al., 2019). Moreover, early-life maternal care has been shown to affect the pup’s physical and functional development in a very broad sense (Caldji et al., 2000; Meaney et al., 2000; Braungart-Rieker et al., 2001; Shin et al., 2008; Curley and Champagne, 2015).

Establishing effective bidirectional communication does not only require that the pup is able to signal distress effectively, but also that the dam is able to perceive, process and respond accurately and timely to these cues (Shin et al., 2008; Bornstein et al., 2017). The separation-induced USV test has been used to assess the quantity and quality of pup USVs after separation from its dam and litter (Hahn and Lavooy, 2005), but it is essentially a unidirectional behavioral assay that focusses on the pup. On the other hand, assays such as the pup retrieval test (PRT) or USV playback tests center on maternal behaviors, such as search and retrieval (Sewell, 1970; Smotherman et al., 1974; Ehret and Haack, 1982; Ehret, 1992, 2005). Some studies implemented both unidirectional procedures separately, but assessed statistical association afterward (Wöhr and Schwarting, 2008; Bowers et al., 2013; Abuaish et al., 2020). Combining both procedures in one behavioral assay has several advantages. First, the behavioral readouts can be sampled in a single assay, which reduces workload, and microenvironmental variability, originating from differences in animal transportation and handling, for example (Sukoff Rizzo and Silverman, 2016; Ey et al., 2020). Second, communication and social competence can be investigated as a bidirectional process in the same animals (Vogel et al., 2019). Third, the complex interaction between deficits in dam and pup can be investigated (Kelly et al., 2000). The latter is particularly important in rodent models of disorders with early-life communication deficits, such as autism or fetal alcohol syndrome (Kelly et al., 2000; Bosque Ortiz et al., 2022). Therefore, we present BAMBI (Bidirectional Automated Mother-pup Behavioral Interaction test), a combined, automated approach to assess early-life communicative bidirectionality in laboratory mice. The automated PRT, as described in Winters et al. (2022), was expanded with simultaneous recording and automated detection of pup USVs.

## Materials and methods

### Animal housing and breeding

C57BL/6J mice from Janvier Labs (Le Genest-Saint-Isle, France) and the KU Leuven Animal Facility (Leuven, Belgium) were time-specifically bred and kept at a 12/12-h light-dark cycle (lights on at 7 a.m.), with *ad libitum* water and food in conditioned rooms (22°C, humidity 30%). The morning after mating was considered as gestational day (GD) 0.5. On GD0.5, dams were housed individually for the remainder of the pregnancy and pregnancies were confirmed between GD7.5 and GD10.5 by recording weight evolution based on Heyne et al. (2015). All experimental procedures were approved by the Animal Ethics Committee of KU Leuven (P028/2018), in accordance with European Community Council Directive 86/609/EEC, the ARRIVE guidelines and the ILAR Guide to the Care and Use of Experimental Animals.

### Experimental groups

In compliance with the reduction principle, mice for the present methodological work were obtained from an independently designed pharmacological study, in which pregnant dams were injected with valproic acid sodium salt (VPA) in order to generate pups representing a neurodevelopmental model of autism. Pups were pharmacologically treated to attempt a rescue of the behavioral impairment. More specifically, pregnant dams (*N* = 44) received a single subcutaneous injection with 60 mg/ml VPA (Sigma Aldrich, Taufkirchen, Germany) dissolved in saline solution on GD12.5. The day of birth was considered as PND0. To standardize nest composition, nests were culled to six pups on PND0 and every nest needed to have at least four pups with both sexes present. These restrictions resulted in 29 dams with viable progeny and a total of 156 pups for further testing. Pups were subcutaneously injected daily from P1-7 with a low (0.5 mg/kg) or a high (2 mg/kg) dose of THIQ (N-[(1R)-1-[(4-Chlorophenyl)methyl]-2-[4-cyclohexyl-4-(1H-1,2,4-trazol-1-ylmethyl)-1-piperidinyl]-2-oxoethyl]-1,2,3,4-tetrahydro-3-isoquinolinecarboxamide; Bio-techne, Abingdon, UK) or a PBS-DMSO control vehicle [doses adapted from Mastinu et al. (2018)]. THIQ was dissolved in PBS and 3.5% DMSO. In total, nine litters (48 pups) were injected with low THIQ dose, 10 l (50 pups) with a high THIQ dose and 10 l (58 pups) with the PBS-DMSO control vehicle. The pharmacological effects are not the focus of the present study and will be described in a separate study. In the current study the aim is to present a proof of principle demonstration of the feasibility and validity of a new automated method for behavioral testing of early life mother-pup bidirectional interactions. Since pharmacological effects were not relevant for the present study, we employed a general linear model (GLM) in which drug effect was set as fixed factor, in order to correct for drug effects (see Calculation of parameters and statistics), and the three drug groups (low-dose THIQ, high-dose THIQ and vehicle) were pooled into one group. For the present study, animals were divided into the following groups: Dams (*n* = 29) and pups (*n* = 156). For the pup sex effect analysis, animals were divided into three groups: dams (*n* = 29), male pups (*n* = 72) and female pups (*n* = 84). For the subsequent analyses investigating the general behavioral interactions between mother and pups, we corrected pup sex effects through a GLM model in which pup sex was set as fixed factor and we pooled male and female pups together. Mice were tested at five time-points: pup postnatal day (PND) 5, 7, 9, 11, and 13.

### Pup retrieval test (PRT) protocol

**FIGURE 1:**
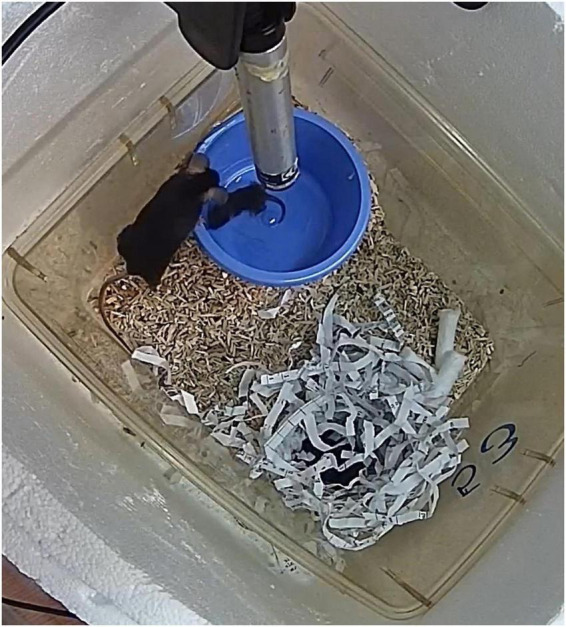
Image of the BAMBI testing setup. BAMBI test was performed in the home-cage and included a cup on PND7-13 to prevent the pups from crawling back into the nest. An ultrasound microphone was placed approximately 5 cm above the test pup’s corner in order to minimize interference from USVs emitted by the pups in the nest (in the opposite corner). A video camera was mounted above the home-cage.

The PRT was performed as described previously by Winters et al. (2022). Briefly, the test is performed in the home-cage which is placed inside a Styrofoam box (Figure 1; 370 mm × 300 mm × 330 mm) to create a visually isolated environment. A single pup was removed from the nest and placed in a clean, glass cup pre-heated to 35°C using a heating pad. A trial was started by a beep when the dam was on the nesting site. Hereafter, the pup was placed in the furthest corner from the nest. Trials had a maximum duration of 100 s after the beep, and when the pup was not retrieved within this time, it was returned to the nest by the experimenter. On PND7-13, since pups had developed more mature motor skills, they were kept from crawling back into the nest by placing them in a cup (Figure 1; 90 mm diameter and 55 mm height) as described by Esposito et al. (2019). Per dam, the PRT was repeated six times on PND5, and due to practical limitations four times per dam on PND7-13. For all test ages, pup sex was counterbalanced per dam and pups were not marked during this test to avoid odor interference. Pups thus could not be identified, meaning that a pup might have been tested more than once. Maternal trial sequence was defined as the order of trials within a dam on a specific testing day. During each trial, PRT performance was scored by the experimenter performing the test (two experimenters in total) for latency to retrieval (s) and retrieval success (0 = not retrieved; 1 = retrieved).

### Ultrasonic vocalization recording and pre-processing

**FIGURE 2:**
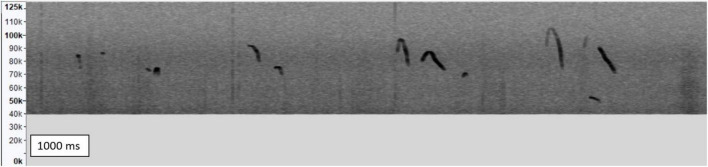
Exemplary spectrogram of ultrasonic vocalizations emitted by pups. Frequency (Hz) is shown on the y-axis over a fixed time of 1,000 ms on the x-axis.

Pup USVs were recorded using an ultrasound microphone (Dodotronic Ultramic UM250K, Rome, Italy) connected to a personal computer equipped with Avisoft SASLab Lite software (Avisoft, Bioacoustics, Berlin, Germany). The microphone was placed approximately 5 cm above the pups’ corner or cup to minimize interference by USVs emitted by the pups in the nest. USVs were recorded for 100 s, with a sampling rate of 250 kHz and 16 bits. Audacity^®^ open-source software (Version 3.1.3)^1^ was used to remove DC (direct current) offset and a equalization (EQ) curve filter was used to remove all signal below 40 kHz (Figure 2).

### Synchronization of USV and behavioral pup retrieval recording

A Foscam C2 IP-camera (EUport, Wageningen) was mounted over the home-cage to record maternal behavior. Per dam, one video was recorded including six PRT trials on PND5 or four PRT trials on PND7-13. A PRT trial started after the dam was back on the nest, and a beep, manually played on a smartphone, introduced the placement of the pup in the furthest corner of the home-cage. Synchronization of behavioral and audio data was done by identifying the beep using frame-precision Shotcut software (version 22.10.25, Meltytech, LLC).^2^ This means video or audio signals can be split precisely per individual frame. Here, videos were recorded using a frame rate of 30 frames per second (fps) and videos were split before the first frame in which the beep was audible. The end of the video was defined 100 s after the first frame with an audible beep. Similarly, the audio recordings were recorded in Avisoft with a sampling rate of 250 kHz, whereas Shotcut was used to remove signal before the beep using a frame rate of 25 fps. Again, the start of the recording was defined as the sampling point before the first fragment in which the beep was audible. For USV recordings, the end was not defined as Avisoft automatically ended sampling after 100 s.

### Automated detection of pup USVs using deep audio segmenter

Ultrasonic vocalization detection was performed using a custom-build model with Deep Audio Segmenter (DAS; 29). DAS 0.26.7 was installed in an Anaconda environment with Python 3.9.13 and training was performed using the DAS notebook on Google Colaboratory [COLAB; (Steinfath et al., 2021)]. Thirty-two audio recordings were pseudo-randomly selected across two different experiments at five ages (postnatal day 5, 7, 9, 11, and 13) and both sexes. Across all recordings, 2,189 pup vocalizations were manually annotated as segments which is equivalent to 67.7 s of pup USVs. Per audio recording, 80% of the annotated USVs were assigned to the training dataset, 10% to the testing dataset and 10% to the validation dataset. A pup USV network was trained in Google COLAB and structural training parameters were chosen based on Steinfath et al. (2021) and can be found in Supplementary File 1. DAS automatically stops training as the validation loss of the model has not improved in 20 epochs (Steinfath et al., 2021). The pup vocalization model did not improve after 44 epochs and performance of this detection model was assessed using precision, recall, F1 scores and overall accuracy. Precision is the percentage of “true cases” per “detected cases.” Recall on the other hand is the percentage of “true cases” per “manually annotated cases.” The F1 score is the harmonic mean of precision and recall.

Data were post-processed for quality control using a custom-build R script to resolve inaccuracies. In the videos, the time was recorded between the first detectable beep segment and the first frame where the hand of the researcher was completely out of the setup after placing the pup in it. All detected USVs were removed from the recording during this time interval.

### Expanded body part tracking

The resulting dataset included 212 frames and was used to re-train the original network from Winters et al. (2022). DeepLabCut 2.2b8 [DLC; (Mathis et al., 2018)] was installed in an Anaconda environment with Python 3.7.7 on a laptop equipped with an Intel Core i5-8350U CPU and 8 GM RAM and Windows 10 64-bit operating system. Training, evaluation and analysis of the expanded model was performed using DLC in Google COLAB.^3^

### Learning strategy and performance evaluation of the PRT pose estimation model

The PRT dam–pup tracking model developed by Winters et al. (2022) was trained to track only PND5 pups in a home-cage without a cup. As C57BL/6J pup body shape changes significantly between PND5 and PND13, and the use of a cup is a significant context change that elicits different maternal poses, the model needed to learn these changes. A two-phase hybrid learning strategy was used similar to Gorssen et al. (2022). In the first phase, fourteen extra single trail video recordings were selected because of their variability in pup age and/or modulated home-cage environment. Fifteen frames per video were extracted using k-means clustering in DLC and labeled manually. Additionally, using the original model 10 extra outlier frames per video were extracted using the DLC “jump” algorithm. Labels in these outlier frames were manually refined and frames were only annotated if both dam and pup were visible. The resulting dataset included 212 frames and the original PRT model was retrained with a 95:5 train/test ratio using the same features as Winters et al. (2022). The model was trained for 47 000 iterations and had a mean pixel error over all body parts of 4.29 px for the training dataset and 14.35 px for the test dataset.

In the second training phase, all original labeled data was combined with the data from the first training phase. The output model from the first training phase was then re-trained using the entire dataset with a 95:5 train/test ratio. After 18 000 iterations, the model had a mean pixel error over all body parts of 6.34 px for the training dataset and 10.11 px for the test dataset. Applying a p cut-off (*p* = 0.10) improved mean pixel error on the training dataset to 5.54 px (or 1.96 mm), and 8.82 px (or 3.13 mm) for the test dataset. Average pixels per millimeter did differ between the original dataset and the data used to extend the dataset. Distance calculation, performed in Simple Behavioral Analysis [SimBA; (Nilsson et al., 2020)] as described by Winters et al. (2022), showed an average of 2.27 (*SD* = 0.3) px/mm in the original dataset, and an average of 2.87 (*SD* = 0.16) px/mm for the newly annotated data.

A custom-build R script was used to post-process the data (quality control) and to estimate retrieval time. First, a time correction was applied to ensure tracking started at the precise moment the pup was placed in the nest. Hereafter, the rolling median (90 frames) of the distance of pup to the nest was calculated to correct for inaccurate tracking in the first seconds of the PRT. The first frame where the rolling median >85 mm was determined. If this was not the first frame, the distance of pup to nest for all previous frames was set to 85 mm, as pups started at least >85 mm from the nest at the start of PRT. Frames with a mean pup tracking probability over all body parts <0.01 were discarded, as these estimates were considered unreliable. Next, a smoothing algorithm was used to approximate the distance of a pup to the nest using the *stat_smooth* function in R (loess method) with a smoothing factor of 0.25. Observed values deviating more than 15 mm from the smoothing estimate were set to missing. After these quality control steps, retrieval time was estimated as the first frame a pup entered the nest.

### Calculation of parameters and statistics

A custom-build R-script (RStudio, Inc., Boston, MA) was used to allow direct comparison between parameters of video and audio analysis. Mean USV duration was calculated as the mean duration of all USVs emitted by the same pup within one trial. USV rate before retrieval was calculated as shown below:


$$U\,S\,V\,r\,a\,t\,e\,\left(\frac{U\,S\,V\,s}{s}\right)=\frac{N\,u\,m\,b\,e\,r\,U\,S\,V\,s\,b\,e\,f\,o\,r\,e\,r\,e\,t\,r\,i\,e\,v\,a\,l}{R\,e\,t\,r\,i\,e\,v\,a\,l\,t\,i\,m\,e\,\left(s\right)}$$

Statistical analyses were performed using the GLM package in R for (binomial) regression models and survival package in R for survival analysis *via* multivariate Cox regression for the trait retrieval success. All models were corrected for USV rate or average USV duration (covariate), sex (fixed effect), day of testing (fixed effect), maternal trial (covariate), and experimental condition (fixed effect).

## Results

### Performance evaluation of DAS audio detection

The USV detection algorithm accomplished an overall accuracy of 99.7%. Noise was predicted with a precision of 99.8 %, a recall of 99.9%, and a F1 score of 99.9%. Pup USVs were predicted with a precision of 94.3%, a recall of 90.2%, and a F1 score of 92.2%.

### Validation of retrieval parameters

To validate the performance of the automated PRT, automatically estimated retrieval times were compared with manual recordings. Retrieval success was estimated with an accuracy of 90.4% (95% *CI* = 87.9–92.5), a sensitivity of 81.0% and specificity of 94.4%. The confusion matrix (Table 1) showed inconsistencies in the prediction of retrieval success of 65 of 670 (9.7%) data entries. After visual inspection, 8 files (Manual: pup not retrieved; Automated: pup retrieved) involved pups walking themselves back into the nest; for 11 files the automated retrieval estimation was more accurate than manual scores; and for 46 files manual scores were more accurate than automated estimations due to tracking errors.

**TABLE 1:** Confusion matrix raw data.

	Manual not retrieved	Manual retrieved
Automated not retrieved	162	27
Automated retrieved	38	451

**FIGURE 3:**
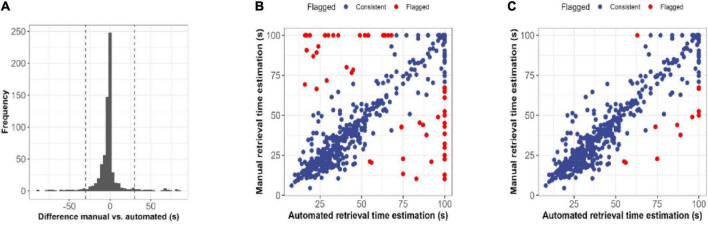
Post-processing quality control of retrieval time estimations. **(A)** Histogram representing the difference in seconds between manual and automated estimations of the retrieval time. Automated estimations of retrieval time were on average 2.4 s faster than manually registered estimations. **(B)** Scatterplot displaying the relationship between raw manual and automated estimations. Differences smaller than 30 s were accepted and shown in blue, whereas differences larger than 30 s were flagged for visual inspection. **(C)** Scatterplot displaying the relationship between corrected manual and automated estimations. After visual inspection of the flagged estimates of figure, the final estimate was either accepted (red) or corrected to the manual estimation.

For estimated retrieval time, Pearson correlations between manual recordings and automated analysis were high (*r* = 0.86). However, estimates using video analysis were on average 2.4 (*SD* = 17.8) seconds faster than manual recordings. Within test day (PND5-13), Pearson correlations ranged between *r* = 0.80 and *r* = 0.92 (PND5: *r* = 0.80; PND7: *r* = 0.80; PND9: *r* = 0.92; PND11: *r* = 0.92; PND13: *r* = 0.86). To establish translatability for the current methodology, manual and automated recordings with a difference larger than 30 s were flagged based on the distribution of differences (Figure 3A). A total of 54 records were flagged of which 31 previously inspected retrieval inconsistencies and the remaining 23 records were visually inspected (Figure 3B). To ensure methodological correctness, 41 automated pup retrieval time estimations were corrected to their manual estimation. Also, pups that walked themselves into the nest were removed from the dataset as bidirectional behavior might be affected. The final dataset is visualized in Figure 3C and the confusion matrix is shown in Table 2. This corrected dataset had an accuracy of 95.1% (95% *CI* = 93.2–96.6), sensitivity of 89.6% and specificity of 97.28%.

**TABLE 2:** Confusion matrix corrected data.

	Manual not retrieved	Manual retrieved
Automated not retrieved	172	13
Automated retrieved	20	465

### Correlations between USV parameters

Correlational analysis showed that the total number of USVs emitted before retrieval was correlated with the USV rate before retrieval (*r* = 0.84; *p* < 0.001), mean USV duration (*r* = 0.44; *p* < 0.001) and first USV event (*r* = −0.30; *p* < 0.001). The same pattern was observed for separate test days (Supplementary Figure 2).

### Repeatability of traits over test days

Repeatability of traits was assessed by looking at the Pearson correlation matrix within a trait over time for the mean value of pups with the same sex within dams (Supplementary Figure 3). For maternal retrieval time, repeatability was generally moderate to high for consecutive test days, significant and consistently positive (*r* = 0.32–0.63; *p* < 0.05–0.001). The correlations suggest that dams who retrieve their pups faster on PND7 generally also will do so on the other days of testing. Pearson correlations between PND5 and the other days of testing were the lowest which might be due to the fact that this was the only day in which the cup paradigm was not used.

Repeatabilities for USV rate and mean USV duration were similarly assessed. Correlations were less pronounced, although most correlations were positive (Supplementary Figures 3, 4). Particularly PND7 gave moderate correlations with the other test days for USV rate (*r* = 0.34–0.50; *p* < 0.05–0.001) and for mean USV duration (*r* = 0.39–0.59; *p* < 0.01–0.001) although not with PND13 data (*r* = 0.12). For latency to first USV emission, no clear pattern was observed although most correlations were positive (Supplementary Figure 5).

**FIGURE 4:**
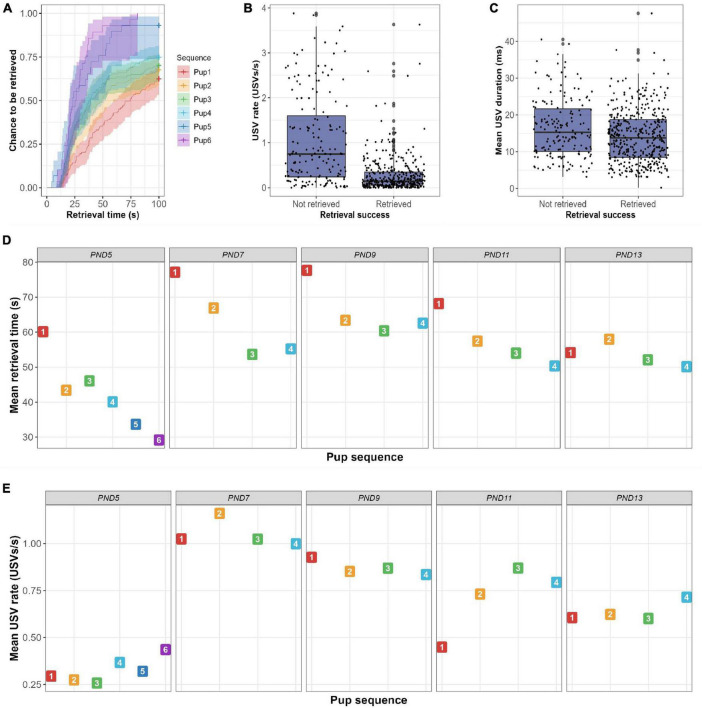
Results of bidirectionality analysis. **(A)** Survival plot representing the estimated probability to be retrieved over time in the PRT per maternal trial. Both retrieval time and chance to be retrieved increased as the maternal trial number increased, suggesting a maternal learning effect. **(B)** Boxplot showing the number of USVs emitted per second when pups are either not retrieved or retrieved. Pups with a higher USV rate had a higher probability not to be retrieved (*p* < 0.001). **(C)** Boxplot showing the mean duration of USVs per pup when pups are either not retrieved or retrieved. Pups with a higher mean USV duration had an increased chance not to be retrieved (*p* < 0.001). **(D)** Mean plot showing the mean retrieval time per maternal trial per day. Although retrieval time decreases significantly for trials within days (*p* < 0.001), the learning effect was not significant between days (*p* = 0.22). **(E)** Mean plot showing the mean USV rate per maternal trial sequence per day. USV rate was not affected by repeated trials (*p* = 0.59), whereas test day significantly did (*p* = 0.02).

### Analysis of pup sex effect

No significant differences between pup sexes were found for USV rate before retrieval (*p* = 0.81), indicating that the number of USVs, proportioned to the retrieval time, was comparable between pup sexes. However, USVs emitted by male pups had a significantly shorter duration compared to the USVs emitted by females (*p* < 0.001). Nevertheless, this did not seem to affect maternal behavior. No significant effect of pup sex on maternal retrieval was observed (*p* = 0.07).

### Analysis of bidirectionality

Correlational analysis of PND5-13 data combined (Supplementary Figure 6), indicated a positive association between pup retrieval time and the amount of USVs the pup emitted (*r* = 0.54; *p* < 0.001), suggesting that pups that vocalized more were retrieved later. Hereafter, we looked at USV emission rate (number of USVs/retrieval time) and the number of USVs recorded during the first 10 s of the test (USVs_10 sec_), as most pups were retrieved after 10 s (5 pups <10 s). This was done to correct for the fact that pups that are retrieved slower, also have more time to emit USVs. However, retrieval time was still positively correlated with USV emission rate (*r* = 0.24; *p* < 0.001). Interestingly, a significantly positive correlation was also found between retrieval time and USVs_10 sec_ (*r* = 0.23; *p* < 0.001).

Hereafter, we performed correlational analyses for each day separately, to exclude the use of the cup and/or age as cofounding variables for these results (Supplementary Figure 6). For total number of USVs emitted before retrieval, moderate, positive correlations were found with retrieval time for all testing days (*r* = 0.45–0.61; *p* < 0.001). This suggests that pups with a higher amount of vocalizations were generally retrieved later. Next, a correction for retrieval time was made by either looking at USV rate or USVs_10 sec_. Here, a significant positive correlation was only found on PND7-9 (*r* = 0.31–0.33; *p* < 0.01) for USV rate and on PND7 and PND13 (*r* = 0.21–0.29; *p* < 0.05) for USVs_10 sec_. It should be noted that non-retrieved pups were assigned a retrieval time of 100 s, which might bias correlations.

**FIGURE 5:**
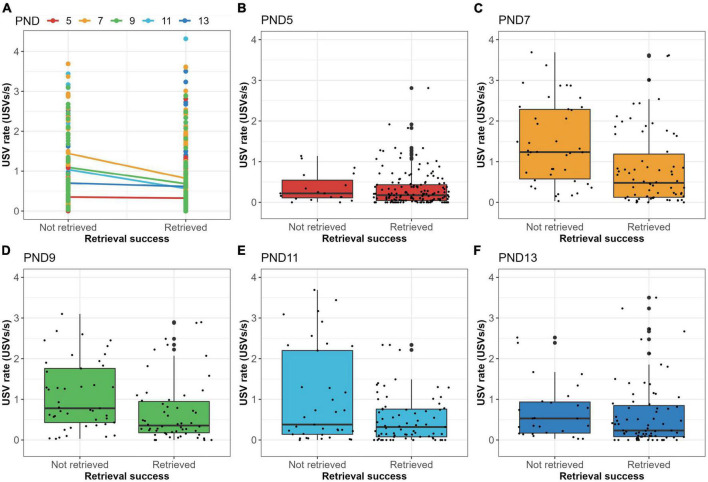
USV rate vs. retrieval success for each test day separately. **(A)** Plot with linear regression of USV rate vs. retrieval success scored as a binary variable for each test day separately. For all test days (PND5-13), USV rate was higher in non-retrieved pups than in retrieved pups, although regression estimates were close to 0 (horizontal regression line) for PND5 and PND13. **(B)** Boxplot showing the USV rate (USVs per second) for pups which are either retrieved or not retrieved on PND5. **(C)** Boxplot showing the USV rate (USVs per second) for pups which are either retrieved or not retrieved on PND7. **(D)** Boxplot showing the USV rate (USVs per second) for pups which are either retrieved or not retrieved on PND9. **(E)** Boxplot showing the USV rate (USVs per second) for pups which are either retrieved or not retrieved on PND11. **(F)** Boxplot showing the USV rate (USVs per second) for pups which are either retrieved or not retrieved on PND13.

The previous results query whether there is a difference in the number of vocalizations emitted by pups that are retrieved and those not retrieved. Binomial regression analysis of PND5-13 data combined, showed that the USV rate was a significant predictor of retrieval success (*HR* = 0.58; *p* < 0.001), which was also indicated by the boxplot (Figure 4B). The hazard ratio (HR) of 0.58 indicates that a USV rate increase of 1 USV/s reduces the probability of being retrieved by 42%. Hereafter, analyses were performed for each day separately, to exclude the use of the cup and/or age as cofounding variables for these results. Figure 5 shows that median USV rate was higher in non-retrieved pups than in retrieved pups, although this difference was small on PND5 and PND13. Binomial regression analyses confirmed these results with negative estimated HR’s on each test day (*HR* = 0.46–0.86) with only significant effects found on PND7, PND9, and PND11. The range of HR between 0.46 and 0.86 over separate test days indicates that a USV rate increase of 1 USV/s reduces the probability of being retrieved by 14–54%.

Furthermore, we wanted to see whether this could be explained by a few poorly retrieving dams (i.e., dams retrieving on fewer than 50% of the trials), such dams were removed from the dataset (*n* = 7 dams). However, the effect of USV rate on retrieval success was still significant after removing poorly retrieving dams (*p* < 0.001). As shown in Supplementary Table 1, some pups (*n* = 67) did not vocalize before retrieval, although 64 of these pups were still retrieved by their dams. Of these 64 trials, 48% occurred on PND5, 20% on PND11, and 19% on PND13. Retrieval without pup vocalization is more common in pups with repeated maternal measurements, i.e., with a later position in maternal trial sequence within a litter (Figure 4A). Moreover, the sequence of maternal trial was found to influence retrieval success significantly (Figure 4A; *P* < 0.001).

The significant effect of maternal trial suggests a learning effect, and as such, provides another possible explanation for the faster retrieval in pups that have a lower vocalization rate. That is, exposing a dam to multiple trials might affect her retrieval behavior and/or might affect pup vocalization rate. However, as shown in Figure 4E, USV rate was not significantly affected by maternal trial (*p* = 0.59), although test day did (*p* = 0.02). Over all days, a maternal learning effect was found to be statistically significant (Figure 4A; *HR* = 1.19; *p* < 0.001). The HR indicates that an increase in maternal trial by one increases the probability of pup retrieval by 19%. As shown in Figure 4D, this maternal learning effect was manifest within repeated trials on the same day (*p* < 0.001), but did not translate between days (*p* = 0.22).

Lastly, the average duration of pup vocalizations was positively correlated with retrieval time (*r* = 0.14; *p* < 0.001), which was most pronounced on PND7-11 (Supplementary Figure 7). Pups emitting USVs with a longer average duration had a lower probability of being retrieved (Figure 4C). The estimated effect in a binomial model was −0.053 (*p* < 0.001) which corresponds with a decreased hazard by a factor of 5% for one extra millisecond of USV.

## Discussion

Bidirectional dam-pup dyad interactions are critical for pup survival. However, most studies investigated dyadic members and behaviors unilaterally (Wöhr and Schwarting, 2008; Abuaish et al., 2020). In the current study, we describe BAMBI (Bidirectional Automated Mother-pup Behavioral Interaction test) to assess bidirectional dam-pup interaction in laboratory mice. This approach combines the automated PRT described by Winters et al. (2022) with synchronous ultrasonic audio recording and subsequent automated USV detection. At first, we demonstrated the transferability of the previously established dam-pup model to a novel experiment with different traits. Further, a model was developed to detect simultaneously recorded pup USVs with high accuracy. Lastly, we applied this methodology on PRT data sampled on PND5, 7, 9, 11, and 13. Indeed, through synchronous video recording of maternal behavior and audio recording of pup vocalizations, BAMBI allowed to test bidirectional early-life mother-pup interactions in an unprecedented way.

We were able to expand the publicly available model (Winters et al., 2022), and optimized its performance for PRT data with different subject and environmental traits such as the inclusion of a cup. We used a hybrid learning strategy to increase variability relatively fast while minimizing bias. This hybrid learning strategy combined manual annotation of k-means selected frames and refinement of outlier frames selected by the DLC “jump” algorithm. In our first attempts, these newly annotated data were added to the annotated dataset of Winters et al. (2022) and retrained. However, pose estimation performance on videos with novel traits was insufficient (data not shown). We hypothesized this might be due to representation bias whereas the original dataset with robust PRT poses on PND5 outweighed the novel dataset with higher pose variability (Krishnan et al., 2021). Therefore, we used a two-step learning approach similar to Gorssen et al. (2022). In a first step, the original model was retrained only with the newly annotated data, whereas in a second step, all annotated data were used to ensure the algorithm performed well on both the original and new data. The automated retrieval estimate can be seen as proof-of-concept and had a high accuracy of 90.4% over all test days. For future research, two remarks on this learning approach should be kept in mind. First, the train and test error after the second retraining step should be interpreted and reported with caution. That is, all data has been used in previous training phases and thus the test data might not be completely new anymore. Second, we found a difference in the average pixels per millimeter when comparing the original dataset and the dataset of the current study. Again, this indicates that the retraining pixel errors should be interpreted with caution.

Further, we were able to develop a model to detect ultrasonic vocalizations in the PRT accurately and automatically using DAS (Steinfath et al., 2021). Despite the wide range of available automated detection options, we chose to work with DAS based on a few selection criteria. First, both the toolbox and its basis software (i.e., Python) are completely open source. Second, the system is versatile which is necessary as this PRT assay intends to investigate early-life communicative deficits, and thus, the emitted vocalizations might not be as expected (Scattoni et al., 2008; Bowers et al., 2013; Ey et al., 2013; Shahrier and Wada, 2018). The system therefore should be easily adaptable and relatively flexible. Third, the system should be able to handle background noise as the PRT is performed in freely moving animals, which are interacting with their environment. As argued in the work of Ey et al. (2020), most available automated systems cannot (yet) handle background noise. However, the main limitation of DAS is that the output is limited to the temporal parameters start and end time of the vocalization. Although this was not a problem for the current study, it is a restriction when investigating communicative deficits. Additional spectrographic output parameters should be an integral part of communicative assessment to fully understand eventual deficits.

An obstacle in the current study was the synchronization of video and audio recordings. Both recording data were sampled using different software and could be synchronized by introduction of a beep at the start of the trial. Although we were able to precisely retrace this beep with frame accuracy, this required an intensive step of data processing. To find its way to standard operational practices, an integrated recording system would significantly reduce human involvement and workload. An exemplary integrated recording system was described in Ey et al. (2020). In this work, behavioral monitoring was done using the Live Mouse Tracker [LMT, (de Chaumont et al., 2019)] system in which synchronized USV sequences were recorded using the Avisoft UltraSoundGate Recording system’s trigger function. The Avisoft burst recording yield an advantage when working with long-term recordings (Ey et al., 2020). However, in the PRT paradigm a maximum time of 100 s is defined and, as previously mentioned, intends to investigate abnormalities in early-life communicative behaviors. The use of burst recordings should be used with caution as it could miss deviant vocalizations and thus could lead to loss of data which cannot be corrected afterward. Other options exist as most Avisoft Ultra Sound Gates have the possibility to connect a TTL cable, which can be used to start ultrasound recording together with another software, e.g., video recording.

Lastly, we demonstrated the effectiveness of our combined methodology by applying it on PRT data sampled on PND5, 7, 9, 11, and 13. It is important to add a note regarding the selection of the study subjects. In compliance with the reduction principle, mice of the present study were obtained from an independently designed pharmacological study. As a consequence, in the absence of controls for experimental disease models, subjects were exposed to VPA and pharmacological treatment, possibly affecting their behavior. Importantly, the aim of the present work was not to investigate pharmacological effects, but rather to present a proof of principle demonstration of the feasibility and validity of a new automated method for behavioral testing of early life mother-pup bidirectional interactions. Nevertheless, in order to address the issue of not being pharmacologically naive, statistical analyses performed in the current study employed a correction for pharmacological treatments as a confounding variable, by using a GLM model in which drug effect was set as a fixed effect, which allowed to pool the different drug groups into a single group (see Experimental groups). Therefore, the general relationships between pup vocalizations and maternal retrieval found in our study can be considered relevant for future research.

We found an association between maternal retrieval success and pup calling behavior. Counterintuitively, we found that pups that were retrieved had a lower call rate during maternal separation than non-retrieved pups (Figure 4B), which was most pronounced on PND7-13. This effect was not caused by certain poorly retrieving mothers, nor testing day. Previous research (D’Amato et al., 2005; Wöhr and Schwarting, 2008) reported a negative relationship between maternal caregiving behaviors and separation-induced pup calling. These studies found that high levels of maternal caregiving behavior in the first days of life lead to reduced numbers of USV later in life, probably because of reduced anxiety. In the same line, maternal carrying has been shown to have soothing effects on pup physiology including cardiac deceleration, immobility response and a reduction of emitted USVs, whereas the absence of this calming response has been reported to hinder maternal retrieval efficacy (Yoshida et al., 2013). Altogether, these findings seemingly go against a robust set of evidence from playback literature which show that pup USVs elicit retrieval behavior (Sewell, 1970; Smotherman et al., 1974; Ehret and Haack, 1982; Ehret, 1992, 2005). Our hypothesis is that USVs do elicit retrieval behavior, but is dependent on a great number of factors (Wöhr et al., 2008) and an excessive amount of USV vocalizations might negatively influence maternal retrieval efficacy. This negative relationship might be due to a miscommunication in the mother-pup dyad. However, further research is necessary to test this hypothesis.

Studies that used maternal retrieval and separation-induced vocalizations separately suggested that these factors might be related. The present simultaneous registrations further confirm and detail this relationship. For example, we found that vocalizations during the first 10 s actually predicted retrieval success, notwithstanding corrections for age and maternal trial sequence. Still, this should not be taken as evidence that pup behavior tunes maternal behavior, as behavioral testing only started on PND5. In our results, we found a peak in USV rate at PND7-9 (Figure 4D), which corresponds with previous findings in literature (Sungur et al., 2016). However, future research might consider earlier time points as communicative fitness might already be affected before PND5 in either quality and/or quantity of vocalizations.

Further, we show that dams subjected to repeated retrieval trials show a significant learning curve within the same test day, although this does not translate to an inter-day effect (Figure 4D). Between PND7 and 9 this might be explained by the introduction of a cup in the home-cage. However, translation is still limited on the other four days that the cup is present. Research has shown that experience improves pup retrieval success (Stolzenberg et al., 2012; Dunlap et al., 2020). Mice tend to use a spatial memory-based strategy when engaged repetitively in pup search and retrieval (Dunlap et al., 2020). Therefore, an overall decrease in retrieval time was to be expected as pups were always placed in the same corner. Additionally, Dunlap et al. (2020) report that retrieval behavior further improves by sensory learning of associated cues. The beep at the start of the trial in the current experiment could have predicted the presence of an separated pup in the home-cage. Our findings seem to contradict the findings of Dunlap et al. (2020) although the number of retrieval repetitions is significantly higher than in our PRT procedure, and the test environment might play a role in the valence of pup stimuli (Stolzenberg et al., 2012). For the interpretation of USVs, this means that the functional relevance of USV emission is particularly high at the beginning. After repeated testing, USV emission seems to be less and less relevant, as evidenced by the fact that retrieval behavior even occurred in the absence of USV emission probably due to maternal learning. However, this maternal learning curve could also be used as a behavioral read-out.

In the present study, we adapted our previous automated home-cage PRT (Winters et al., 2022) and we combined video recording of maternal behavior with synchronous audio recording of pup vocalizations in order to assess bidirectional dam-pup dyadic interaction. Our methodology expands the automated pup retrieval test with automated detection of pups’ ultrasonic vocalizations. Moreover, we validated our results and showed that the number and rate of ultrasonic vocalizations are associated with retrieval success. BAMBI is a promising new automated home-cage behavioral method that can be applied to both basic and preclinical studies on early-life social development.

## Data availability statement

The original contributions presented in this study are included in the article/Supplementary material. All models used for this study are publicly available at: 10.17605/OSF.IO/VEJ4H. Further inquiries can be directed to the corresponding author.

## Ethics statement

This animal study was reviewed and approved by the Animal Ethics Committee of KU Leuven (P028/2018).

## Author contributions

CW and RD’H designed the experimental strategy. CW optimized experimental procedures, labeled the data, and wrote the manuscript with input from WG, MW, and RD’H. CW and WG conceptualized and wrote the code. All authors contributed to the article and approved the submitted version.

## Footnotes

1
https://www.audacityteam.org/

2
www.shotcut.org

3
https://colab.research.google.com/
