# Supplementary material for: BAMBI: A new method for automated assessment of bidirectional early-life interaction between maternal behavior and pup vocalization in mouse dam-pup dyads
Source: Front Behav Neurosci. 2023 Mar 3;17:1139254. doi: 10.3389/fnbeh.2023.1139254 (PMC10020184; doi:10.3389/fnbeh.2023.1139254)
Supplement: Supplementary file 1 [file Data_Sheet_1.PDF]

Supplementary Material

Automated assessment of bidirectional early-life interaction in mouse dam-pup dyads

C. Winters[1,4], W. Gorssen[2], M. Wöhr[3,4,5,6] & R. D'Hooge[1,4]

[1] Laboratory for Biological Psychology, KU Leuven, Tiensestraat 102 – PO Box 3714, 3000, Leuven, Belgium

[2] Department of Biosystems, Center for Animal Breeding and Genetics, KU Leuven, Leuven, Belgium

[3] KU Leuven, Faculty of Psychology and Educational Sciences, Research Unit Brain and Cognition, Laboratory of Biological Psychology, Social and Affective Neuroscience Research Group, Leuven B-3000, Belgium

[4] KU Leuven, Leuven Brain Institute, Leuven B-3000, Belgium

[5]Behavioral Neuroscience, Experimental and Biological Psychology, Faculty of Psychology, Philipps-University Marburg, 35032 Marburg, Germany.

[6]Center for Mind, Brain and Behavior, Philipps-University Marburg, 35032 Marburg, Germany.

*** Correspondence:**
Corresponding Author
carmen.winters@kuleuven.be

1 Supplementary Data

Supplementary File 1. Structural parameters used to train the DAS automated detection network.

```
das.train.train(model_name='tcn_stft',
    data_dir=path_to_data,
    save_dir=path_to_data,
    pre_nb_conv=4,
    pre_nb_dft=33,
    pre_nb_filters=33,
    pre_kernel_size=64,
    nb_hist=8192,
    batch_size=32,
    batch_norm=True,
    kernel_size=16,
    nb_filters=32,
    ignore_boundaries=True,
    verbose=1,
    nb_conv=2,
    learning_rate=0.0005,
    use_separable=[True, True, False, False],
    nb_epoch=100)
```

2 Supplementary Figures and Tables

2.1 Supplementary Tables

Supplementary Table 1. Table including pups that did not vocalize.

	SEQUENCE	RETRIEVED	NOT RETRIEVED
**PND5**		31	2
	Pup 1	*1*	*1*
	Pup 2	*4*	*0*
	Pup 3	*6*	*0*
	Pup 4	*2*	*1*
	Pup 5	*8*	*0*
	Pup 6	*10*	*0*
**PND7**		4	0
	Pup 1	*1*	*0*
	Pup 2	*1*	*0*
	Pup 3	*1*	*0*
	Pup 4	*1*	*0*
**PND9**		4	0
	Pup 1	*0*	*0*
	Pup 2	*1*	*0*
	Pup 3	*1*	*0*
	Pup 4	2	*0*
**PND11**		13	1
	Pup 1	*5*	*0*

	Pup 2	*2*	*1*
	Pup 3	*4*	*0*
	Pup 4	2	*0*
**PND13**	12	0	
	Pup 1	*2*	*0*
	Pup 2	*3*	*1*
	Pup 3	*2*	*0*
	Pup 4	5	*0*

2.2 Supplementary Figures

Supplementary Figure 1. Pairwise correlational plots of total amount of emitted USVs (number USVs), USV rate (USVs/s), average USV duration (s) and latency to first USV (s). Pairwise correlation plots are first given for all test days combined (PND5-13) and then separately for PND5, 7, 9, 11 and 13. Below diagonal the pairwise correlation plot is shown. Above the diagonal the estimated Pearson correlation coefficient is given with significance value. (°p<0.10; *p<0.05; **p<0.01; ***p<0.001)

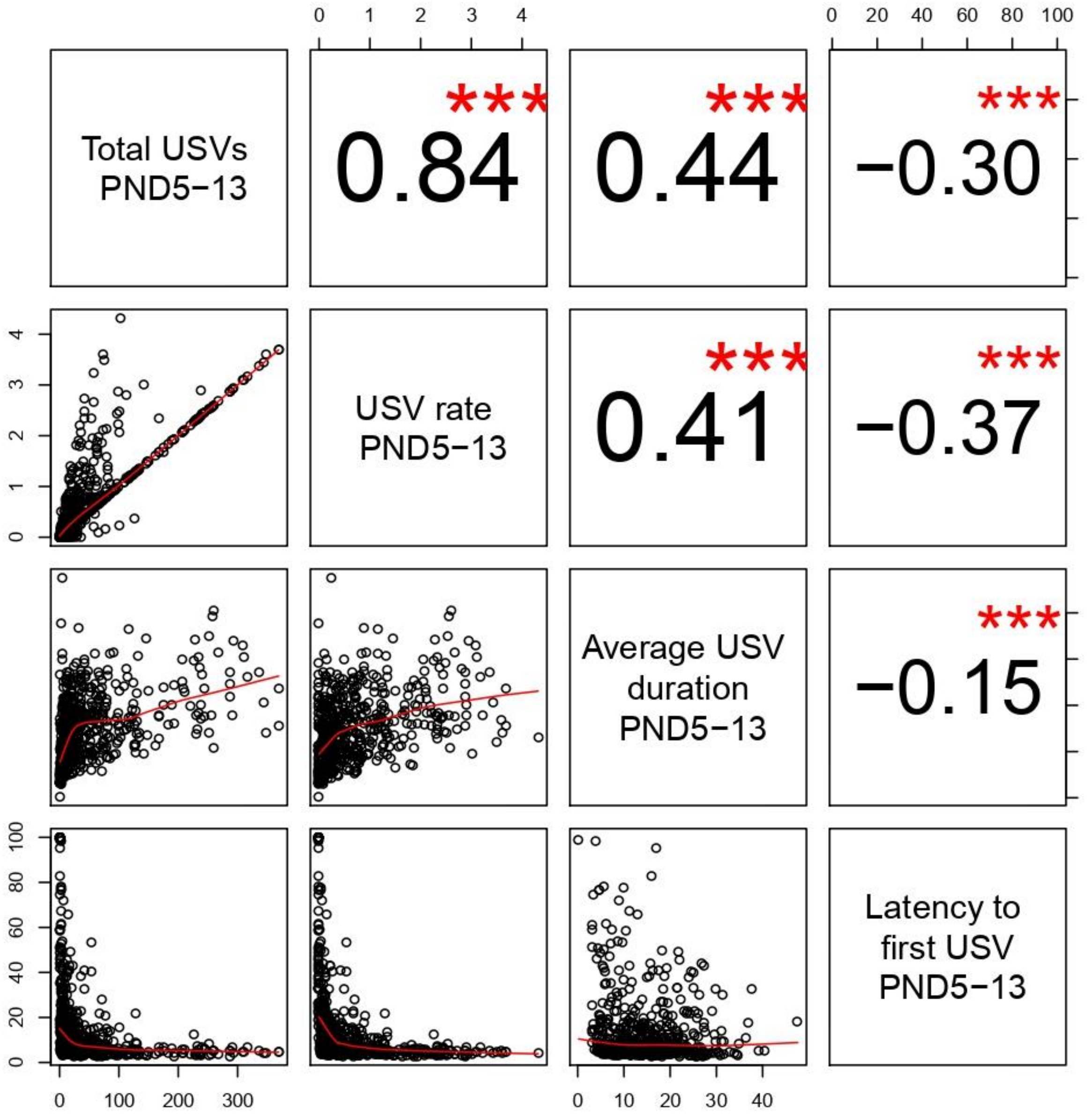

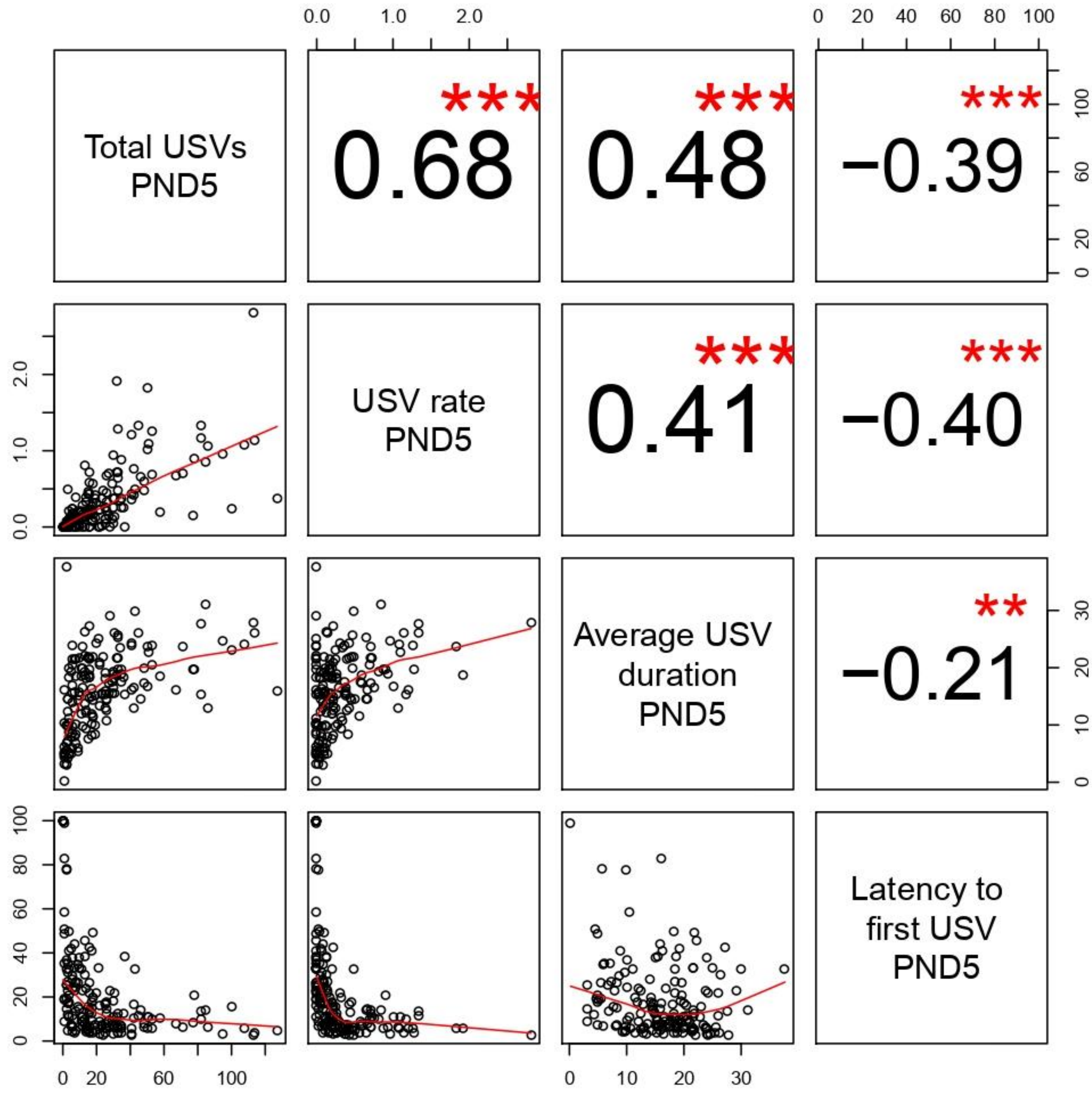
Total USVs PND5
0.68
0.48
−0.39
USV rate PND5
0.41
−0.40
Average USV duration PND5
−0.21
Latency to first USV PND5

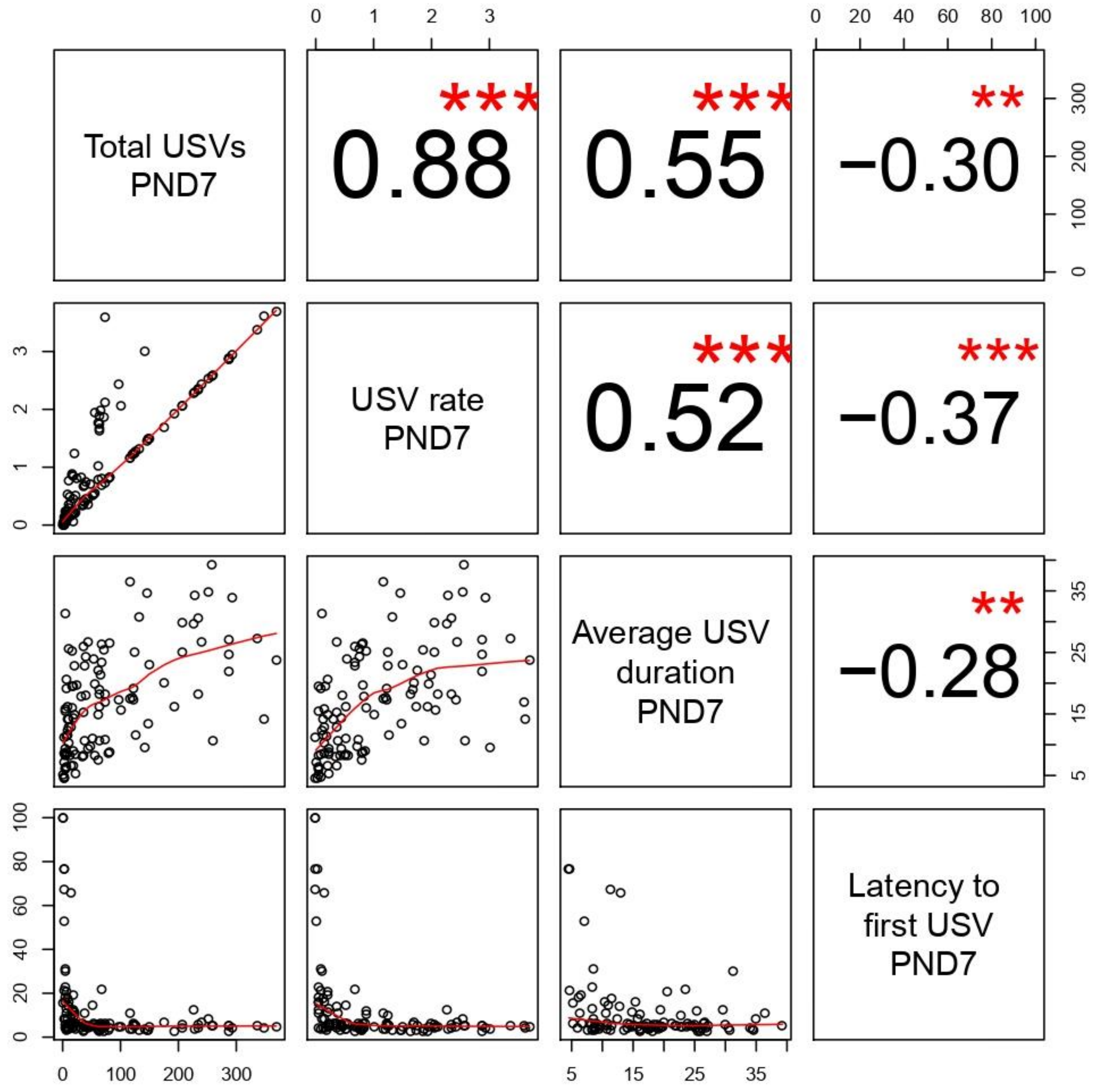
Total USVs PND7
USV rate PND7
Average USV duration PND7
Latency to first USV PND7
***
0.88
***
0.55
**
−0.30
***
0.52
***
−0.37
**
−0.28
0 1 2 3
0 20 40 60 80 100
0 100 200 300
5 15 25 35

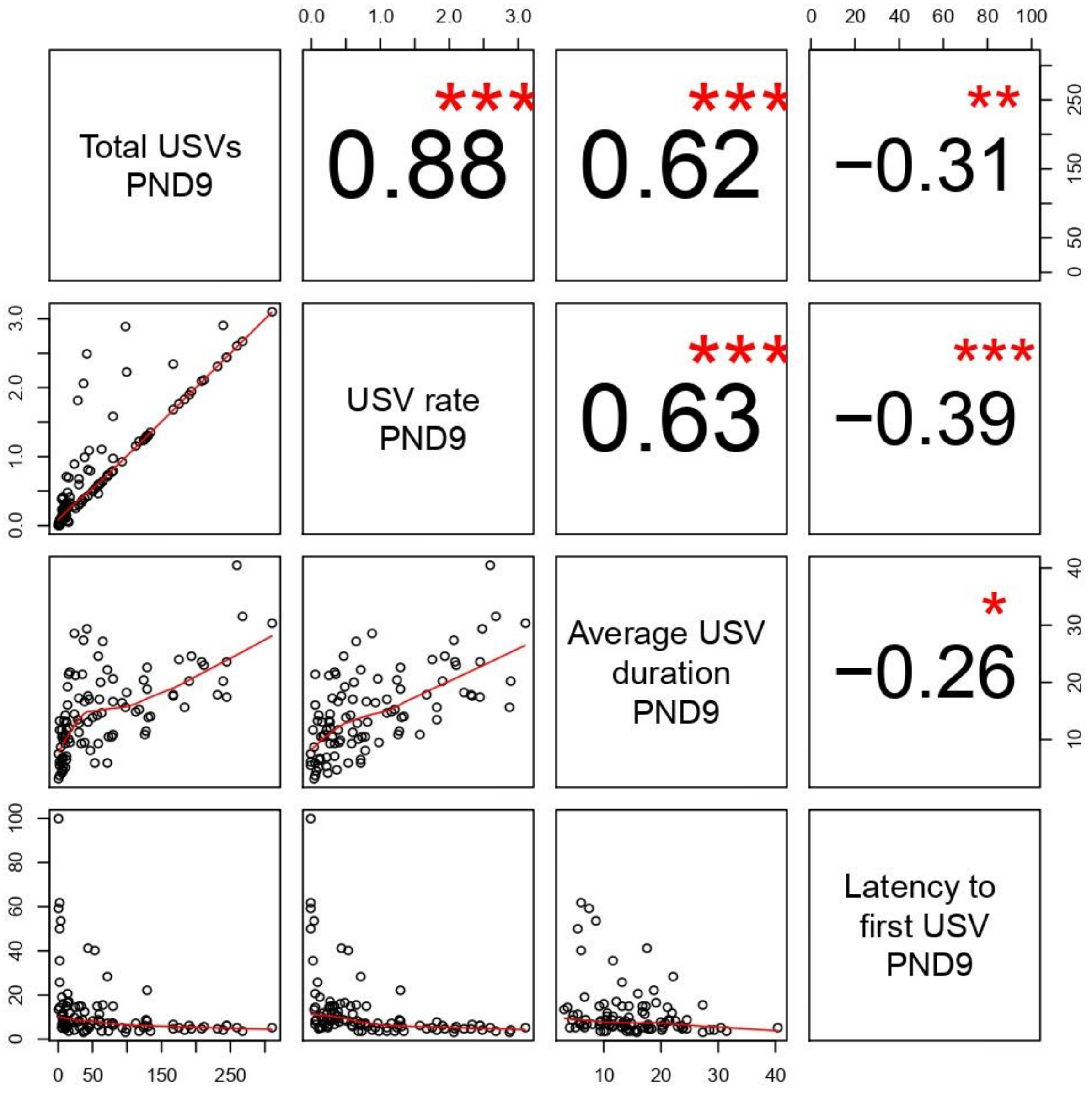
0.0
1.0
2.0
3.0
0
20
40
60
80
100
Total USVs
PND9
***
0.88
***
0.62
**
−0.31
250
150
50
0
3.0
2.0
1.0
0.0
USV rate
PND9
***
0.63
***
−0.39
40
30
20
10
Average USV
duration
PND9
*
−0.26
100
80
60
40
20
0
Latency to
first USV
PND9
0
50
150
250
10
20
30
40

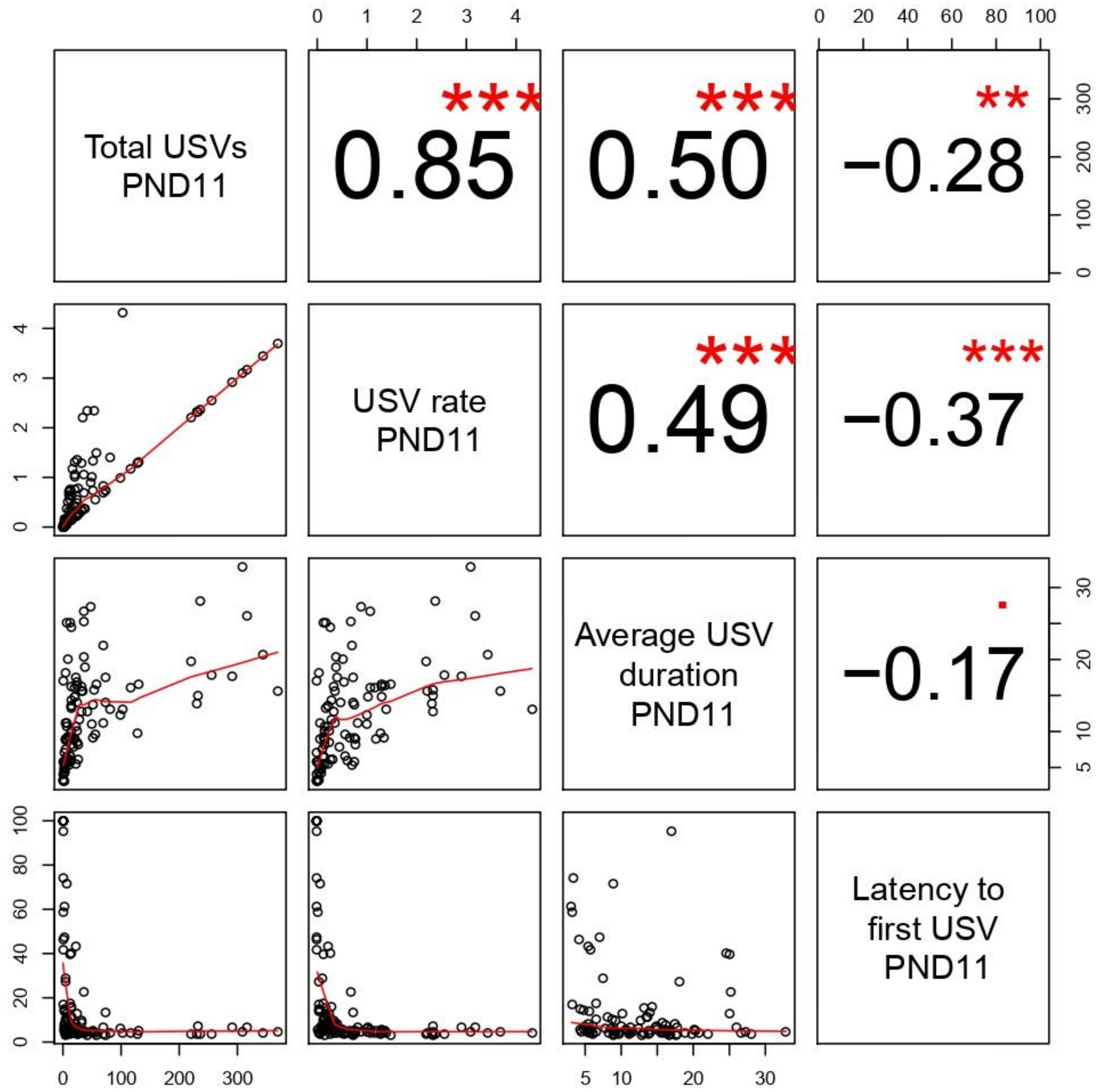

Total USVs
PND11
0.85
***
0.50
***
−0.28
**
USV rate
PND11
0.49
***
−0.37
***
Average USV
duration
PND11
−0.17
.
Latency to
first USV
PND11

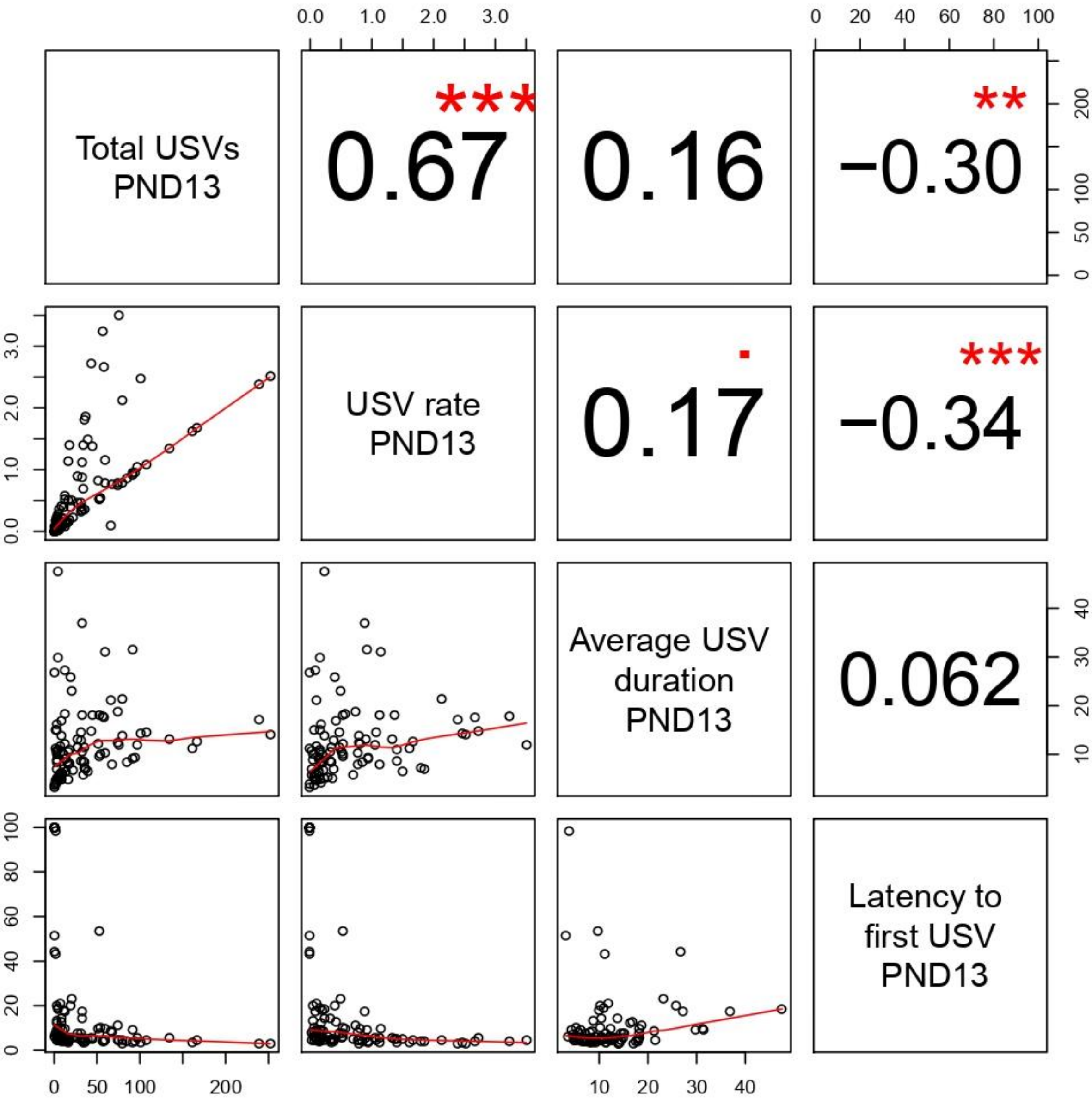

Total USVs PND13
0.67
***
0.16
−0.30
**
USV rate PND13
0.17
.
−0.34
***
Average USV duration PND13
0.062
Latency to first USV PND13

Supplementary Figure 2. Repeatability of maternal retrieval time represented as a pairwise correlational plot of retrieval time (s) over PND5, 7, 9, 11 and 13. Below diagonal the pairwise correlation plot is shown. Above the diagonal the estimated Pearson correlation coefficient is given with significance value. (°p<0.10; *p<0.05; **p<0.01; ***p<0.001)

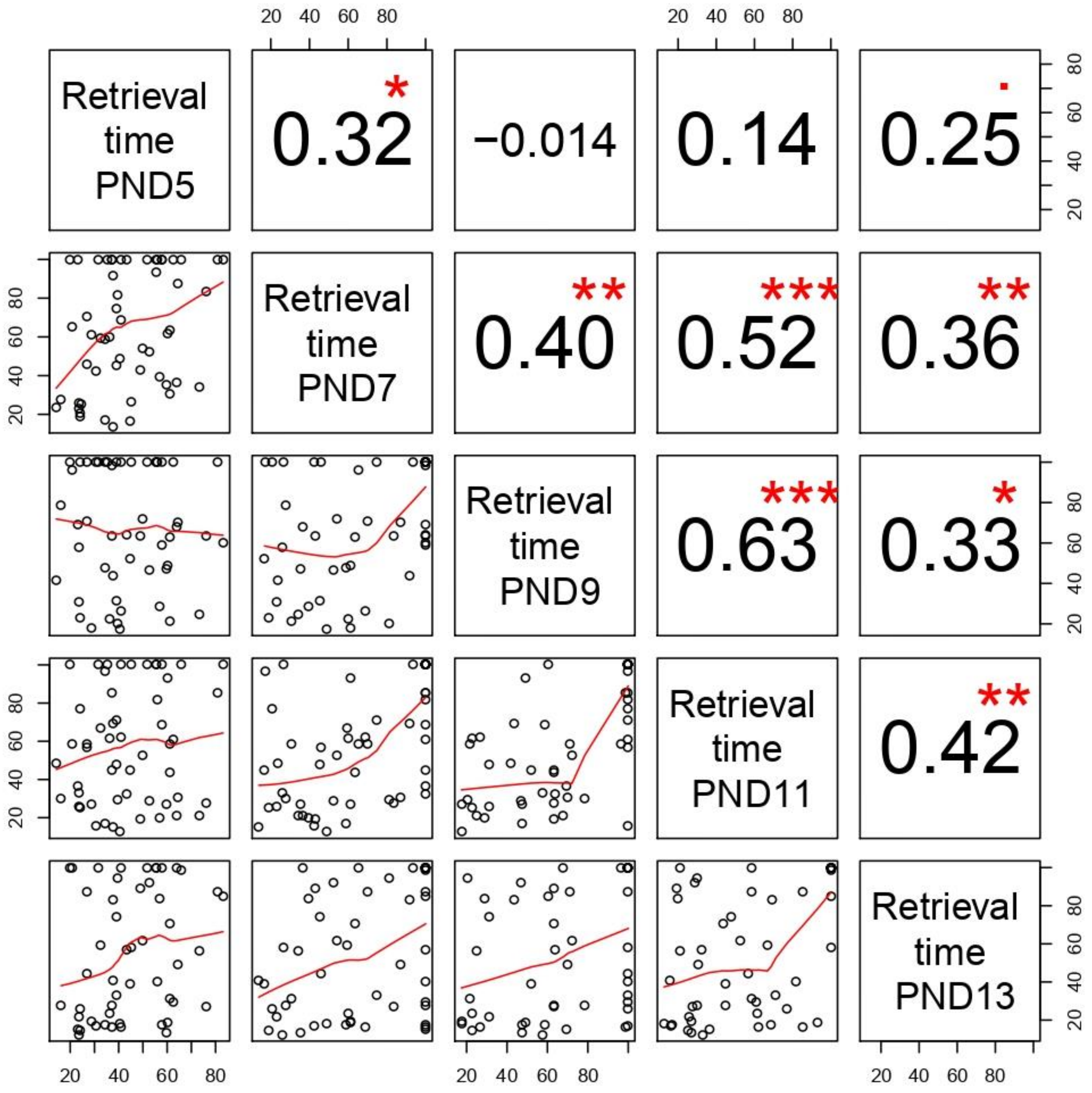

Supplementary Figure 3. Repeatability of USV rate represented as a pairwise correlational plot of USV rate (USVs/s) over PND5, 7, 9, 11 and 13. Below diagonal the pairwise correlation plot is shown. Above the diagonal the estimated Pearson correlation coefficient is given with significance value. (°p<0.10; *p<0.05; **p<0.01; ***p<0.001)

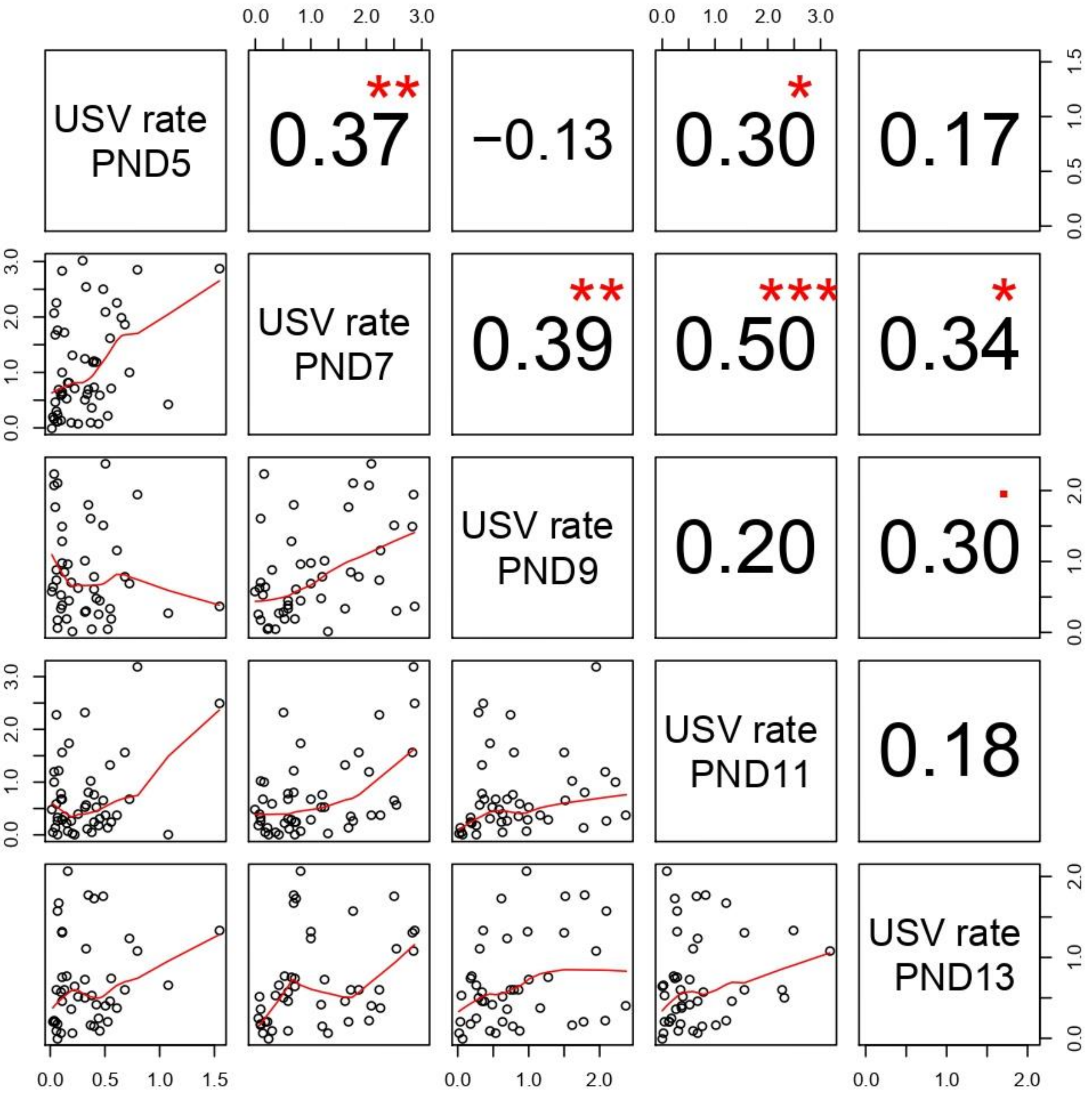

Supplementary Figure 4. Repeatability of average USV duration represented as a pairwise correlational plot of average USV duration (s) over PND5, 7, 9, 11 and 13. Below diagonal the pairwise correlation plot is shown. Above the diagonal the estimated Pearson correlation coefficient is given with significance value. (°p<0.10; *p<0.05; **p<0.01; ***p<0.001)

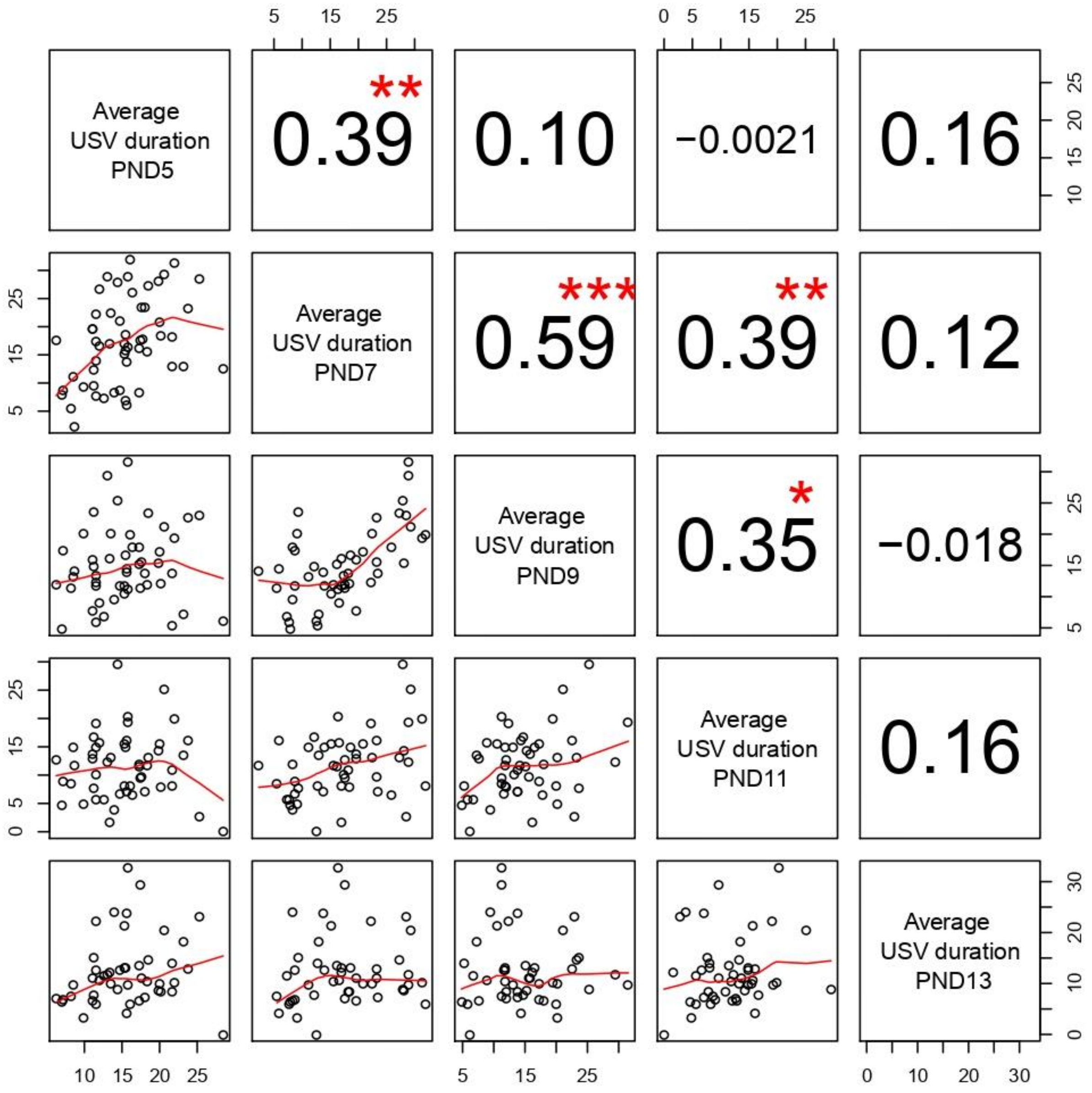

Supplementary Figure 5. Repeatability of latency to first USV represented as a pairwise correlational plot of latency to first USV (s) over PND5, 7, 9, 11 and 13. Below diagonal the pairwise correlation plot is shown. Above the diagonal the estimated Pearson correlation coefficient is given with significance value. (°p<0.10; *p<0.05; **p<0.01; ***p<0.001)

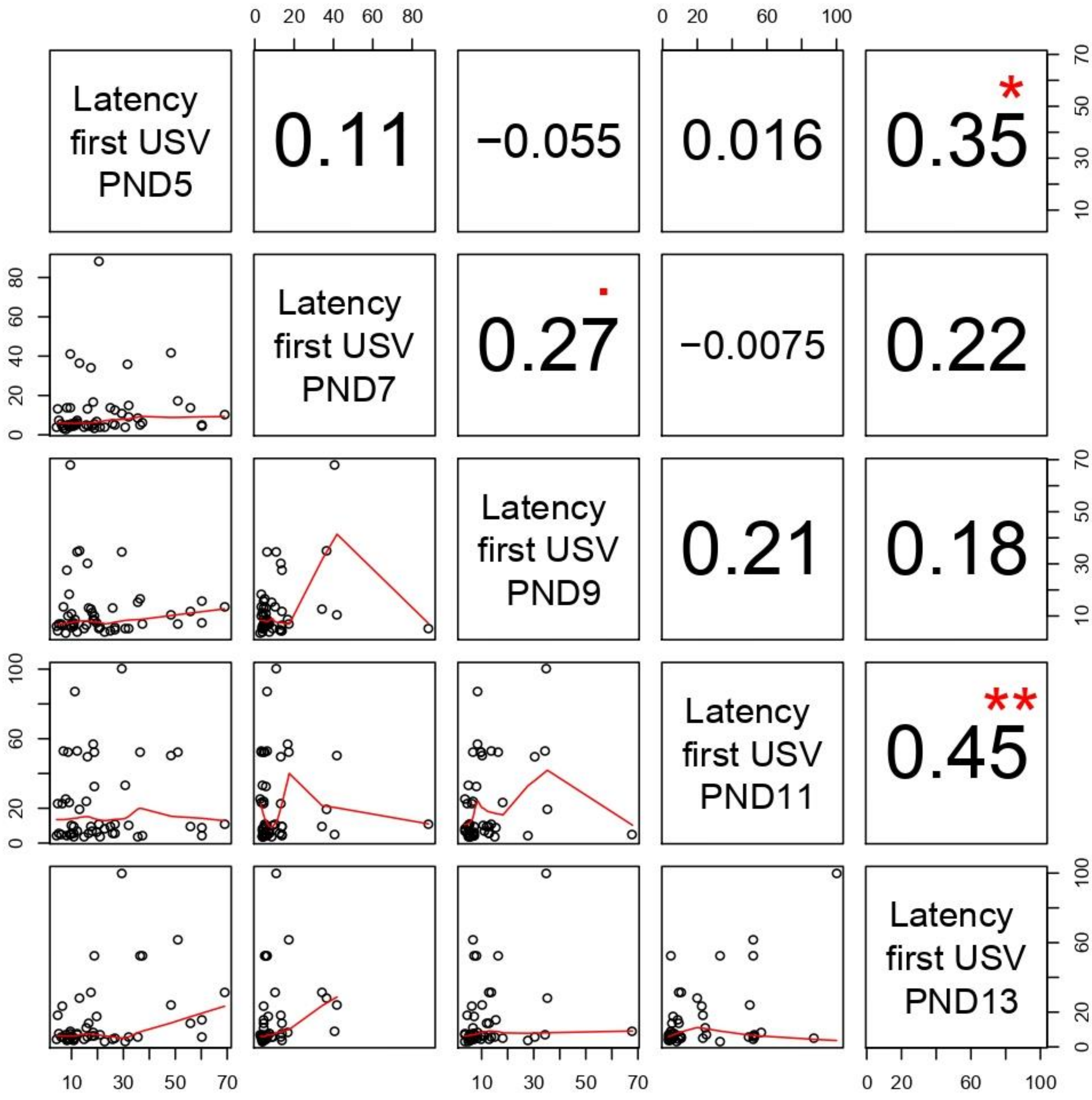

Supplementary Figure 6. Pairwise correlational plots of retrieval time (s), USVs emitted before retrieval (number USVs), USV rate (USVs/s) and USVs emitted in the first 10 seconds of PRT trial (number USVs). Pairwise correlation plots are first given for all test days combined (PND5-13) and then separately for PND5, 7, 9, 11 and 13. Below diagonal the pairwise correlation plot is shown. Above the diagonal the estimated Pearson correlation coefficient is given with significance value. (°p<0.10; *p<0.05; **p<0.01; ***p<0.001)

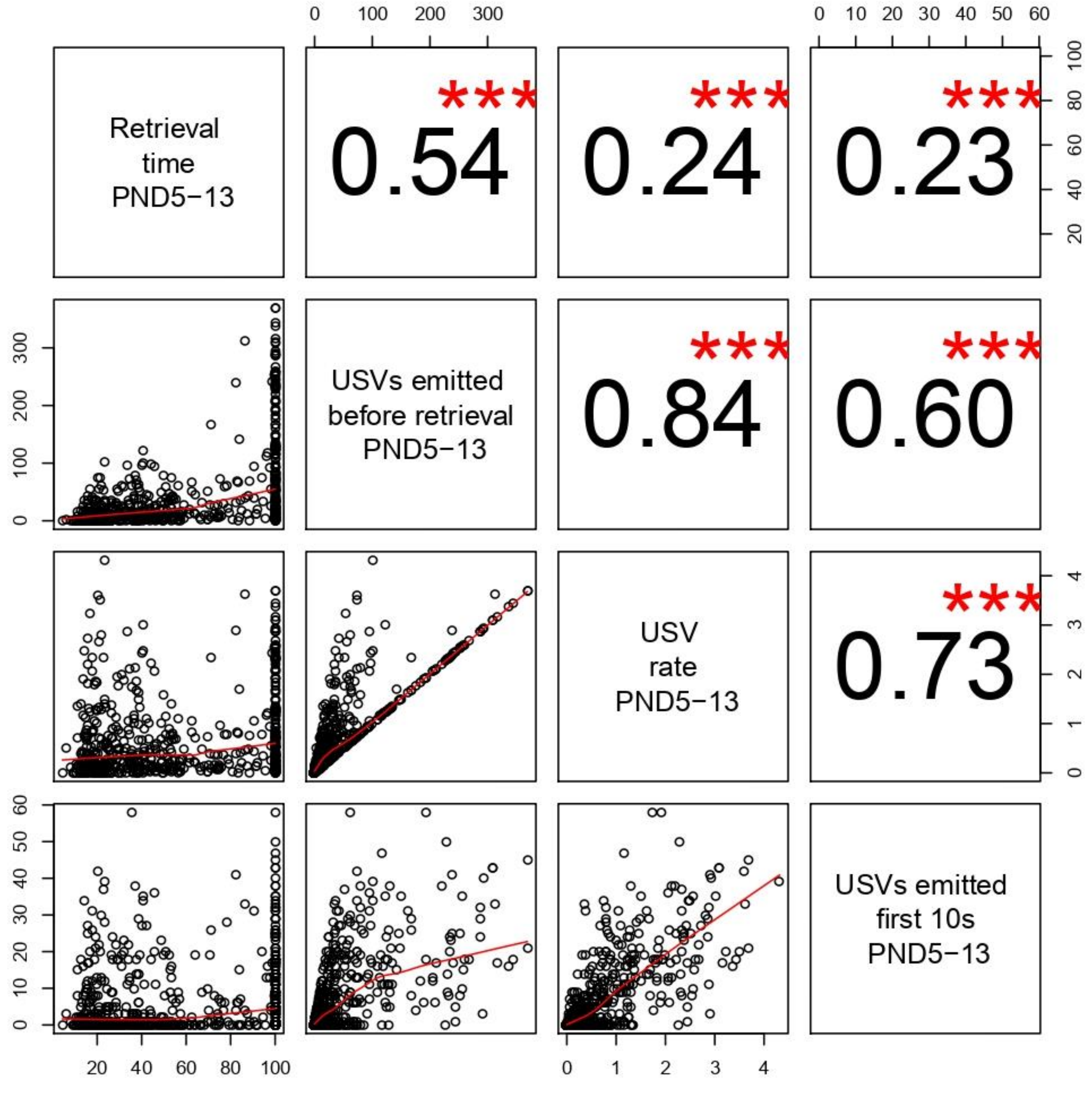

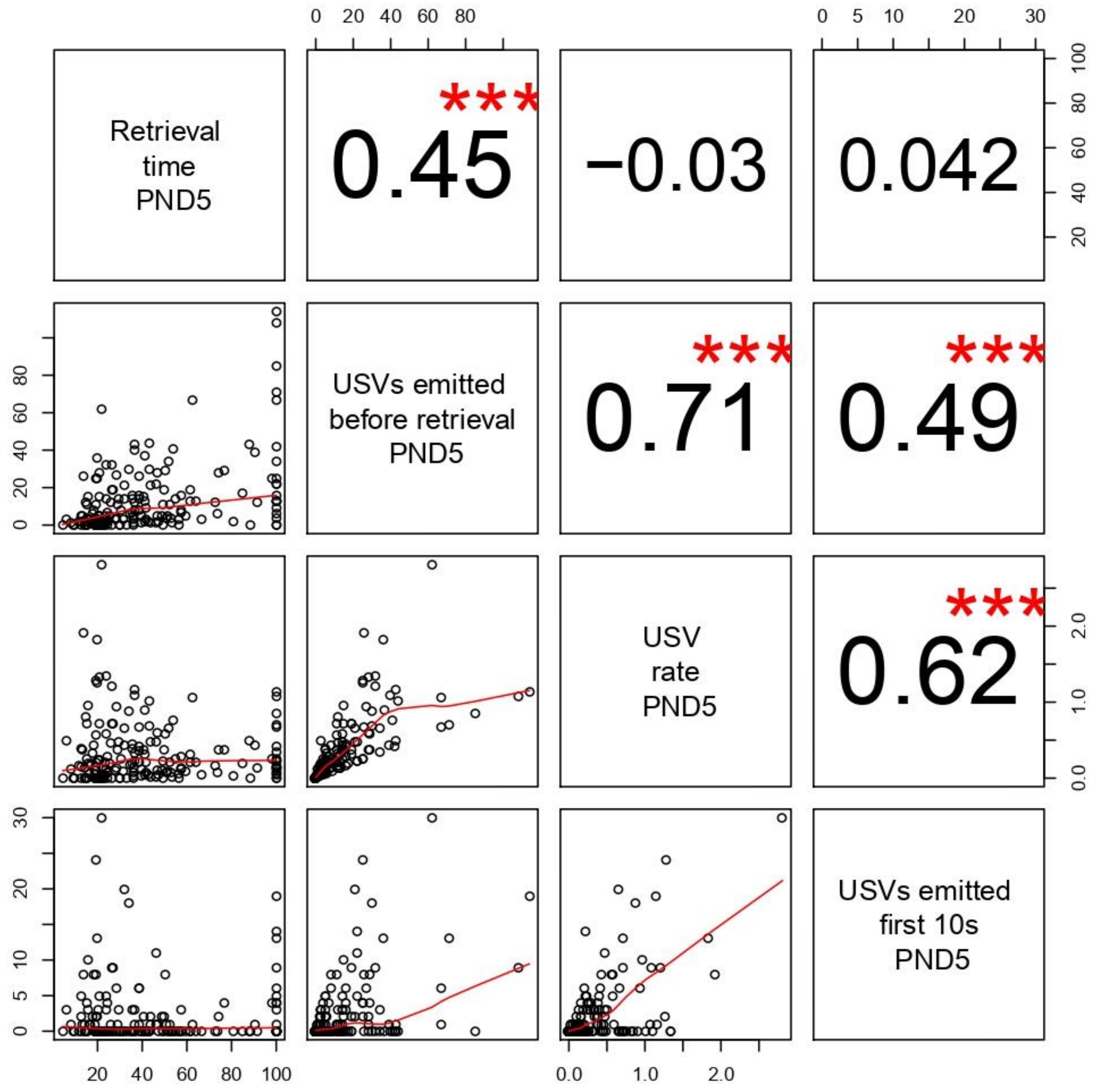
Retrieval time PND5
USVs emitted before retrieval PND5
USV rate PND5
USVs emitted first 10s PND5
0.45 ***
−0.03
0.042
0.71 ***
0.49 ***
0.62 ***

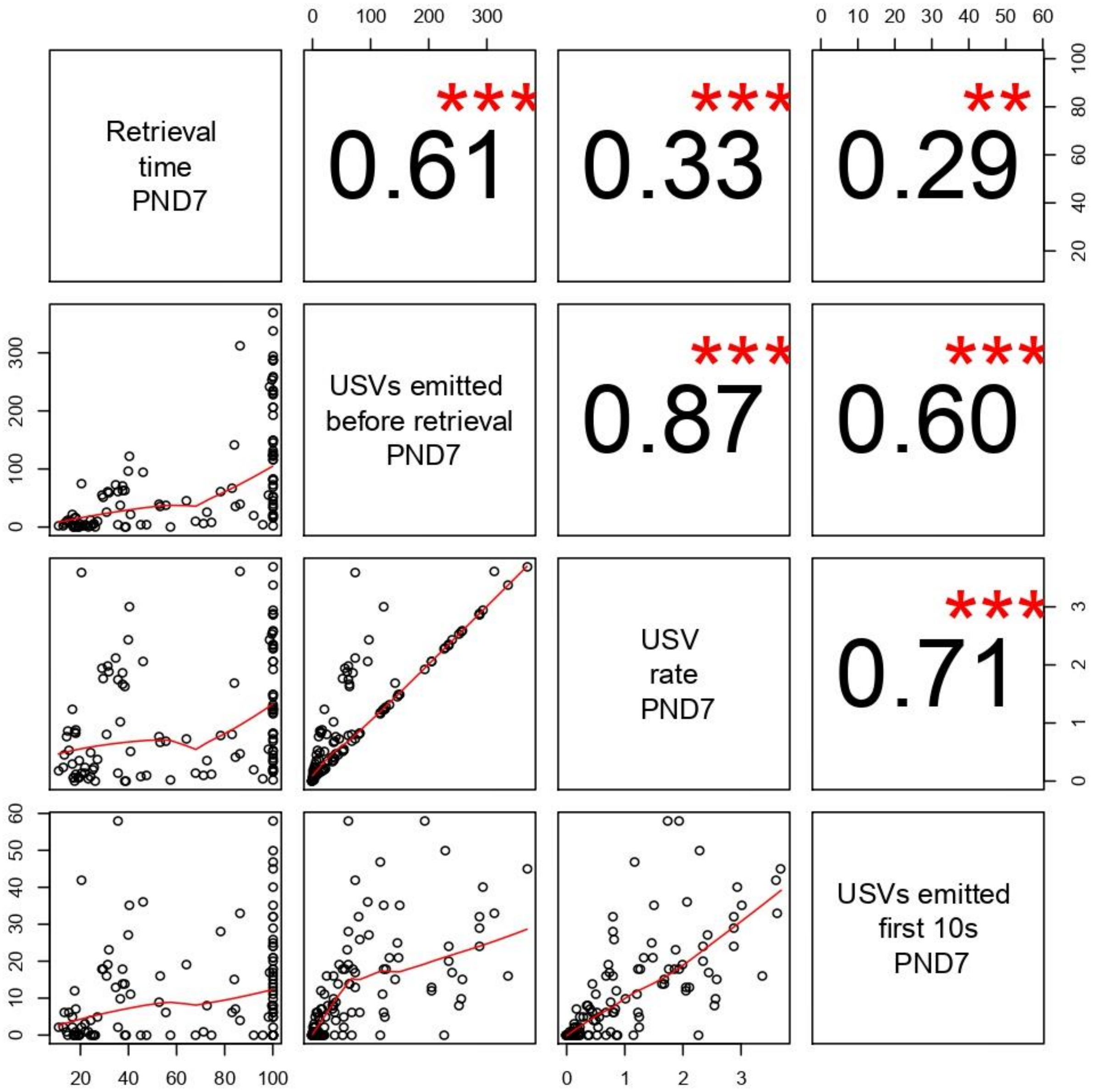
Retrieval time PND7
USVs emitted before retrieval PND7
USV rate PND7
USVs emitted first 10s PND7
0.61
***
0.33
***
0.29
**
0.87
***
0.60
***
0.71
***

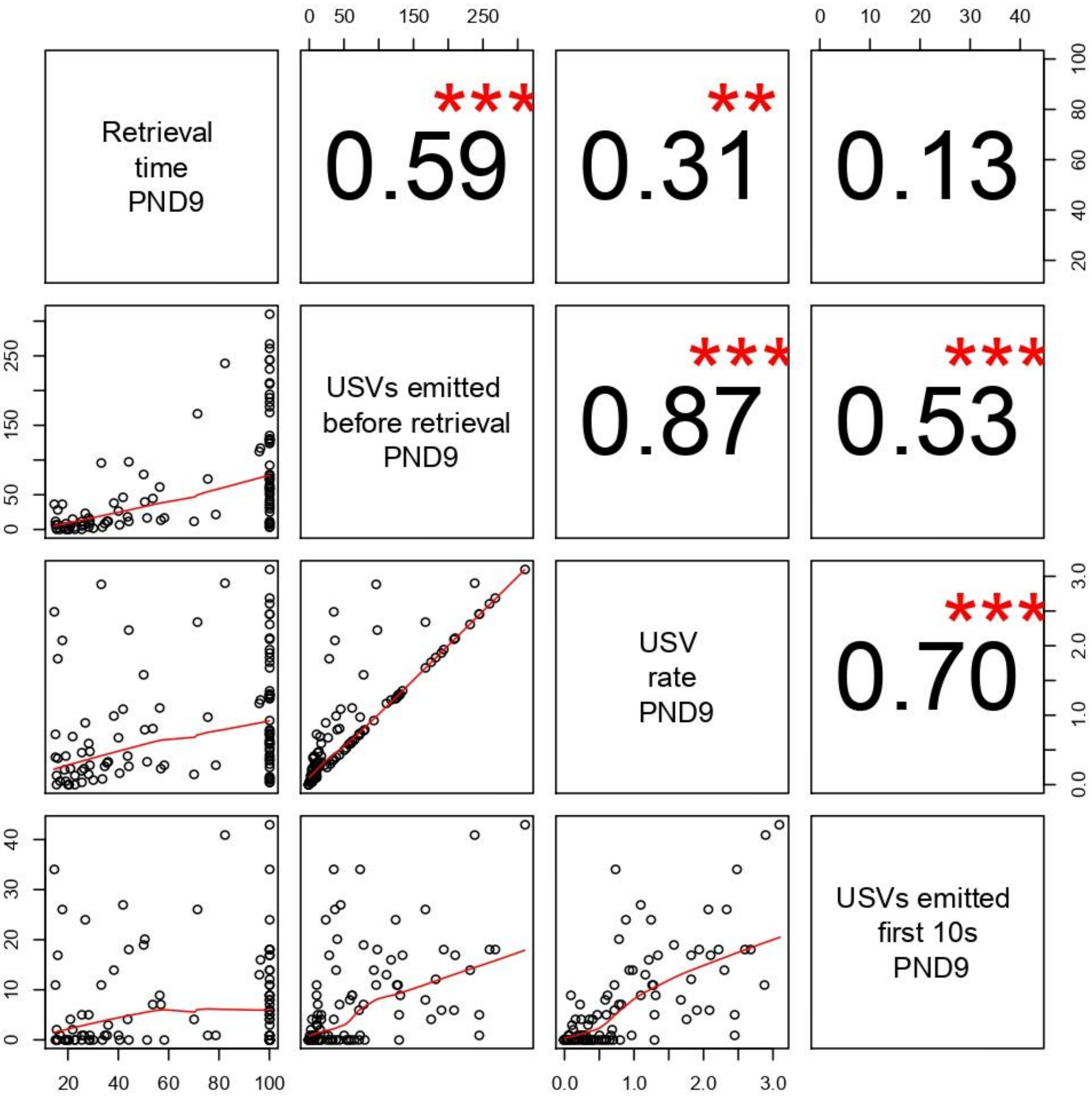
Retrieval time PND9
USVs emitted before retrieval PND9
USV rate PND9
USVs emitted first 10s PND9
0.59 ***
0.31 **
0.13
0.87 ***
0.53 ***
0.70 ***
0 50 150 250
0 10 20 30 40
20 40 60 80 100
0 50 150 250
0.0 1.0 2.0 3.0
0 10 20 30 40
20 40 60 80 100
0.0 1.0 2.0 3.0

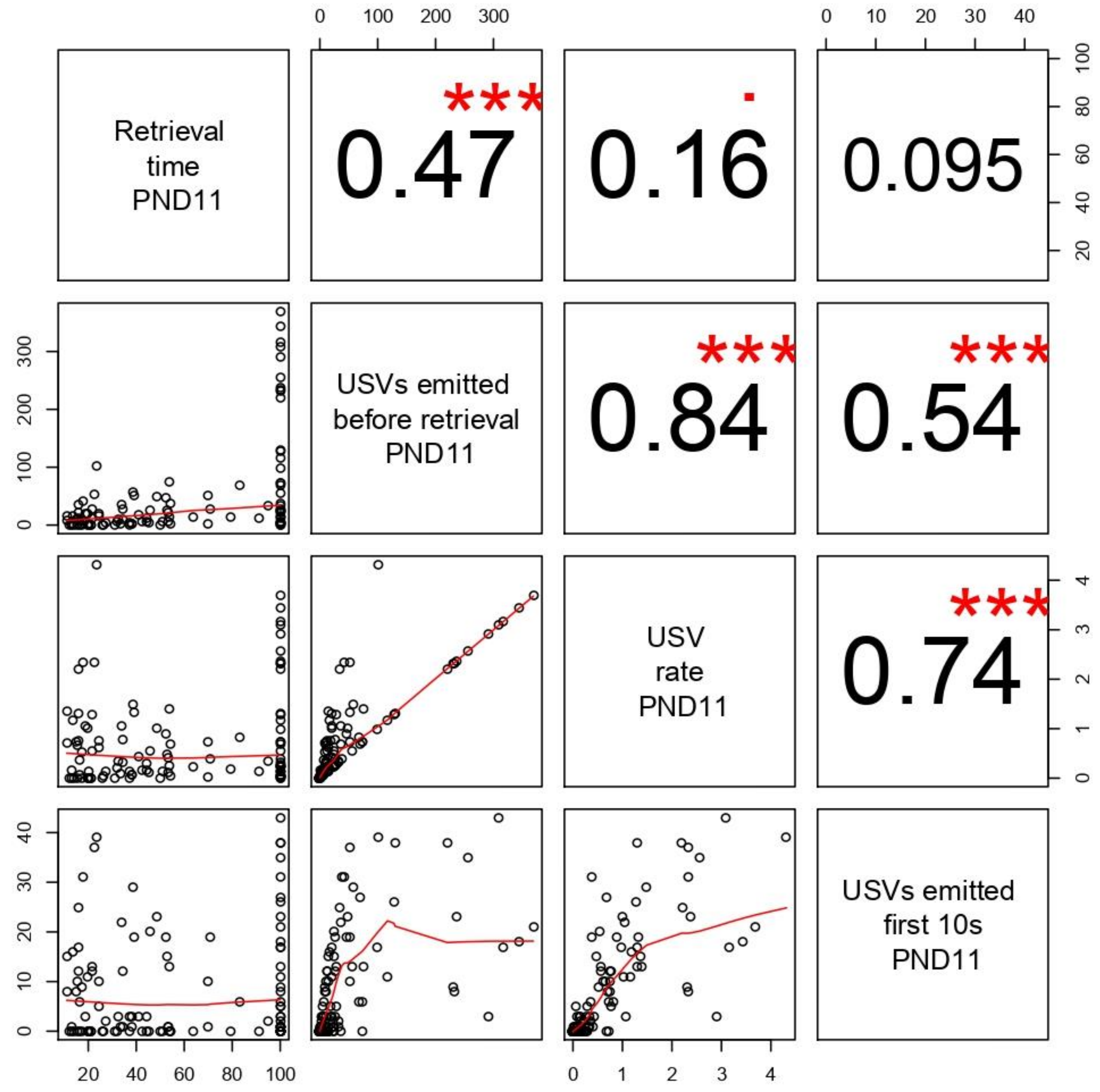

Retrieval time PND11
0.47 ***
0.16 .
0.095
USVs emitted before retrieval PND11
0.84 ***
0.54 ***
USV rate PND11
0.74 ***
USVs emitted first 10s PND11
0 100 200 300
0 10 20 30 40
20 40 60 80 100
0 1 2 3 4

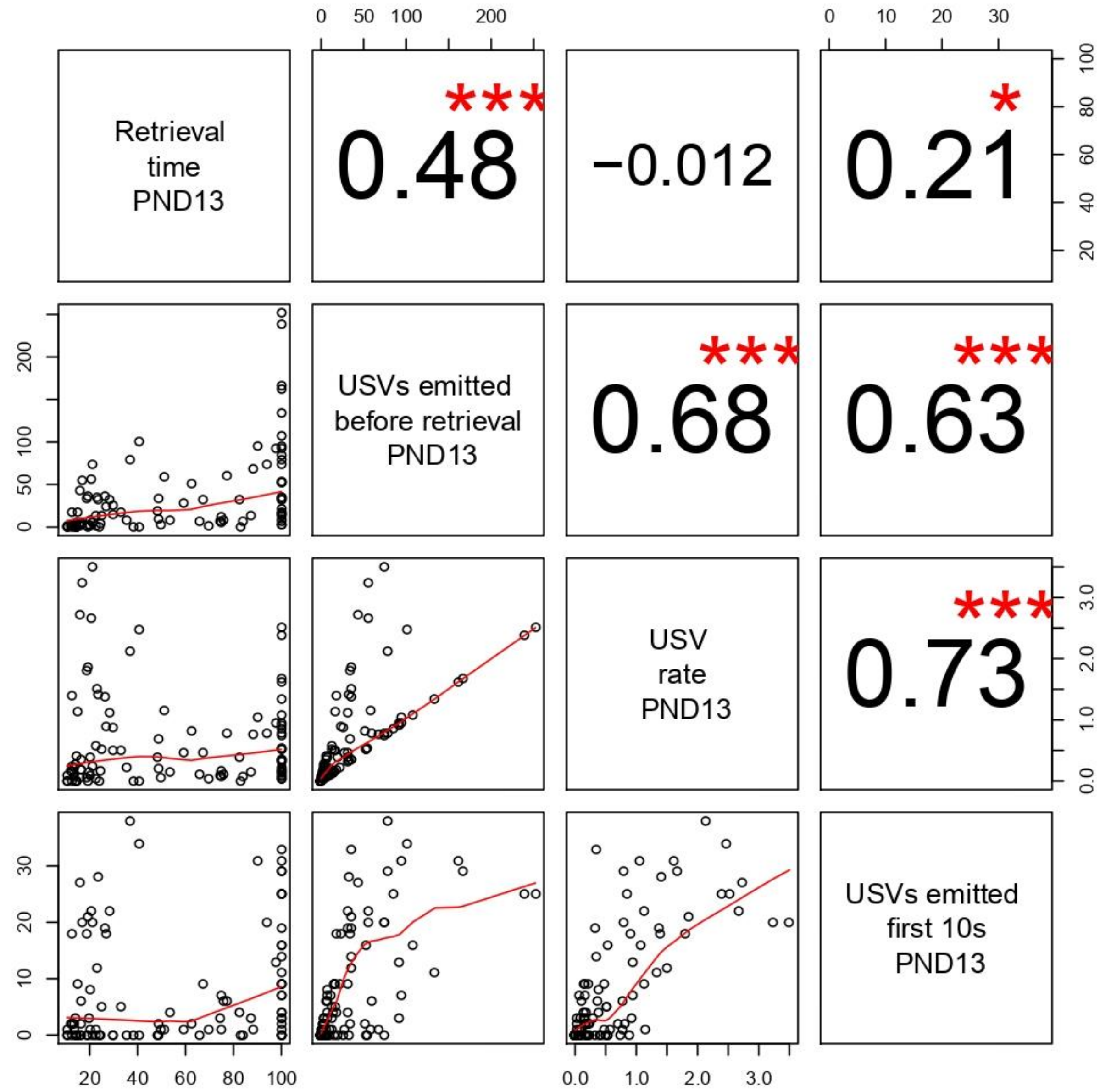
Retrieval time PND13
USVs emitted before retrieval PND13
USV rate PND13
USVs emitted first 10s PND13
0.48 ***
−0.012
0.21 *
0.68 ***
0.63 ***
0.73 ***

Supplementary Figure 7. Pairwise correlational plots of retrieval time (s), average USV duration (s) and sum of USV duration (s). Pairwise correlation plots are first given for all test days combined (PND5-13) and then separately for PND5, 7, 9, 11 and 13. Below diagonal the pairwise correlation plot is shown. Above the diagonal the estimated Pearson correlation coefficient is given with significance value. (°p<0.10; *p<0.05; **p<0.01; ***p<0.001)

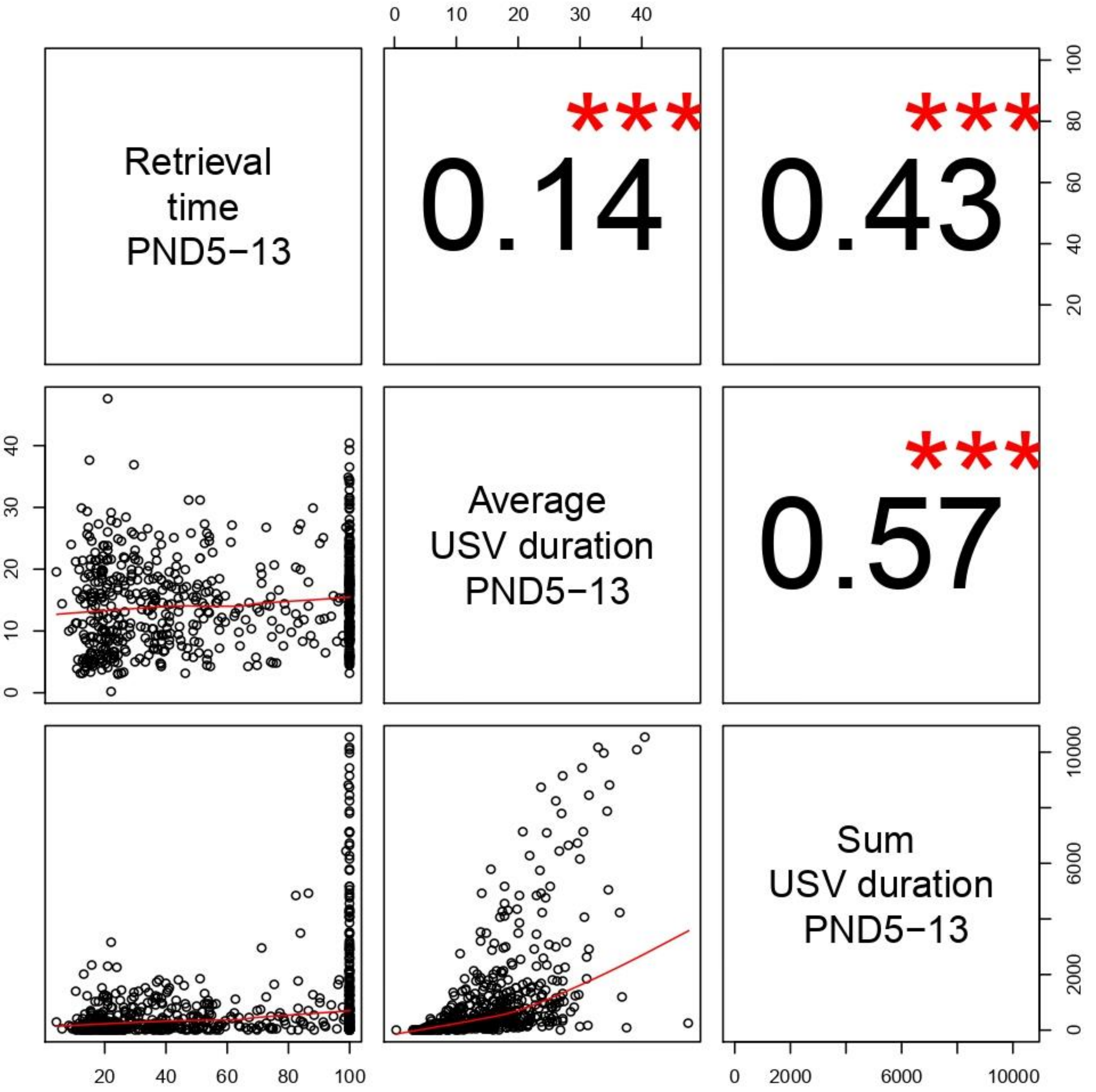

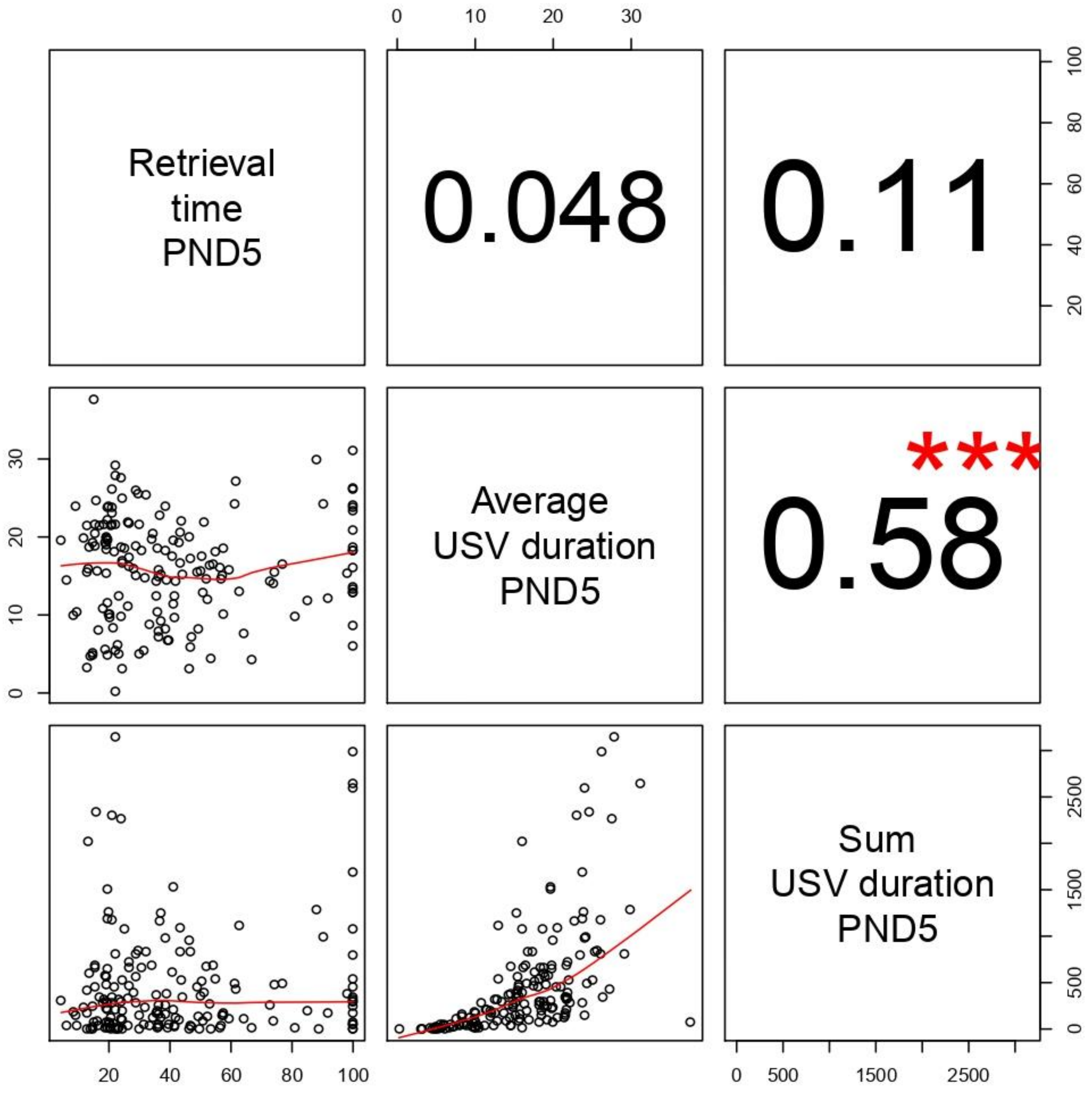

0
10
20
30
100
80
60
40
20
Retrieval
time
PND5
0.048
0.11
30
20
10
0
Average
USV duration
PND5
***
0.58
2500
1500
500
0
Sum
USV duration
PND5
20
40
60
80
100
0
500
1500
2500

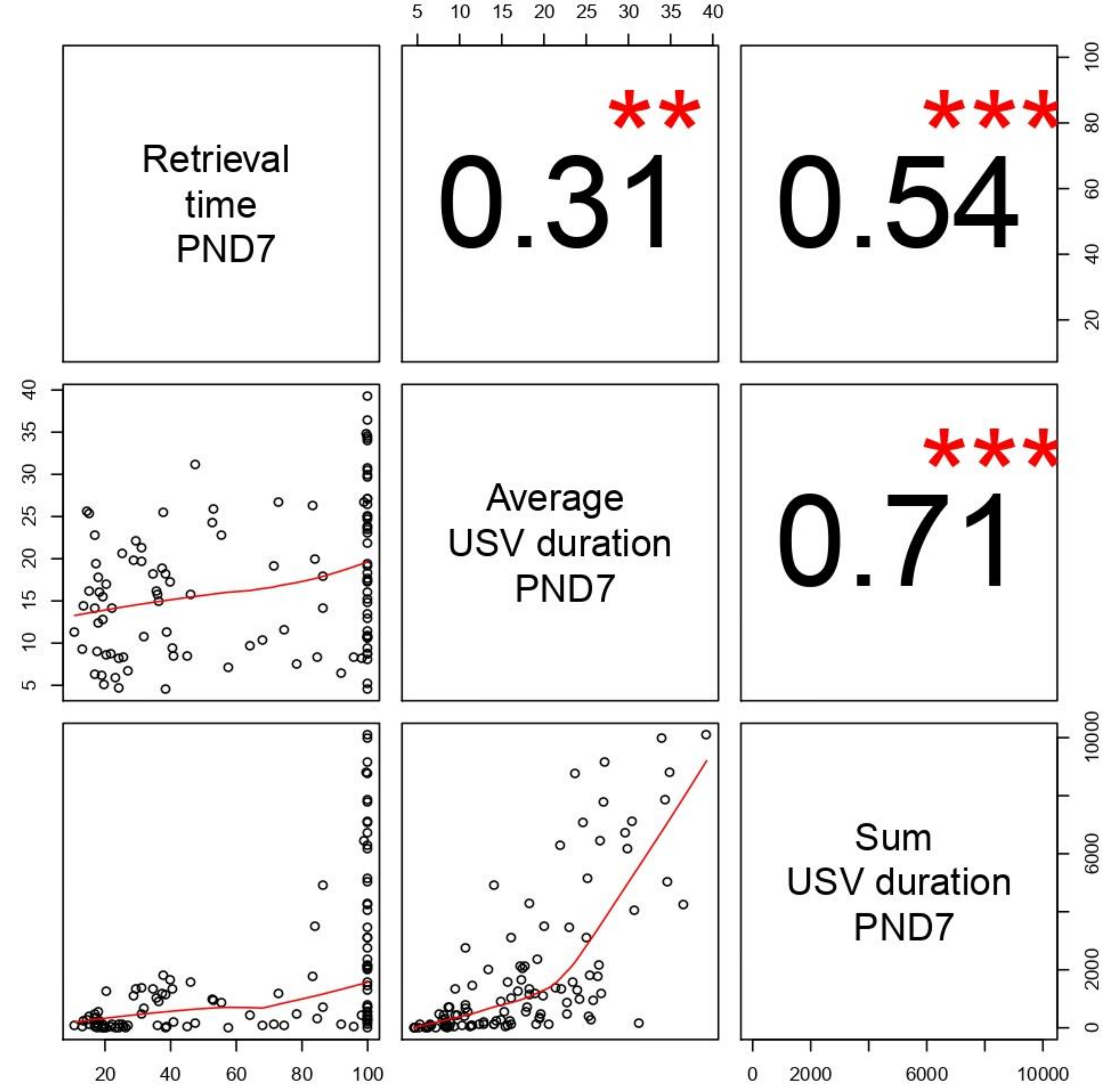
Retrieval time PND7
0.31
**
0.54
***
Average USV duration PND7
0.71
***
Sum USV duration PND7
5 10 15 20 25 30 35 40
20 40 60 80 100
0 2000 6000 10000

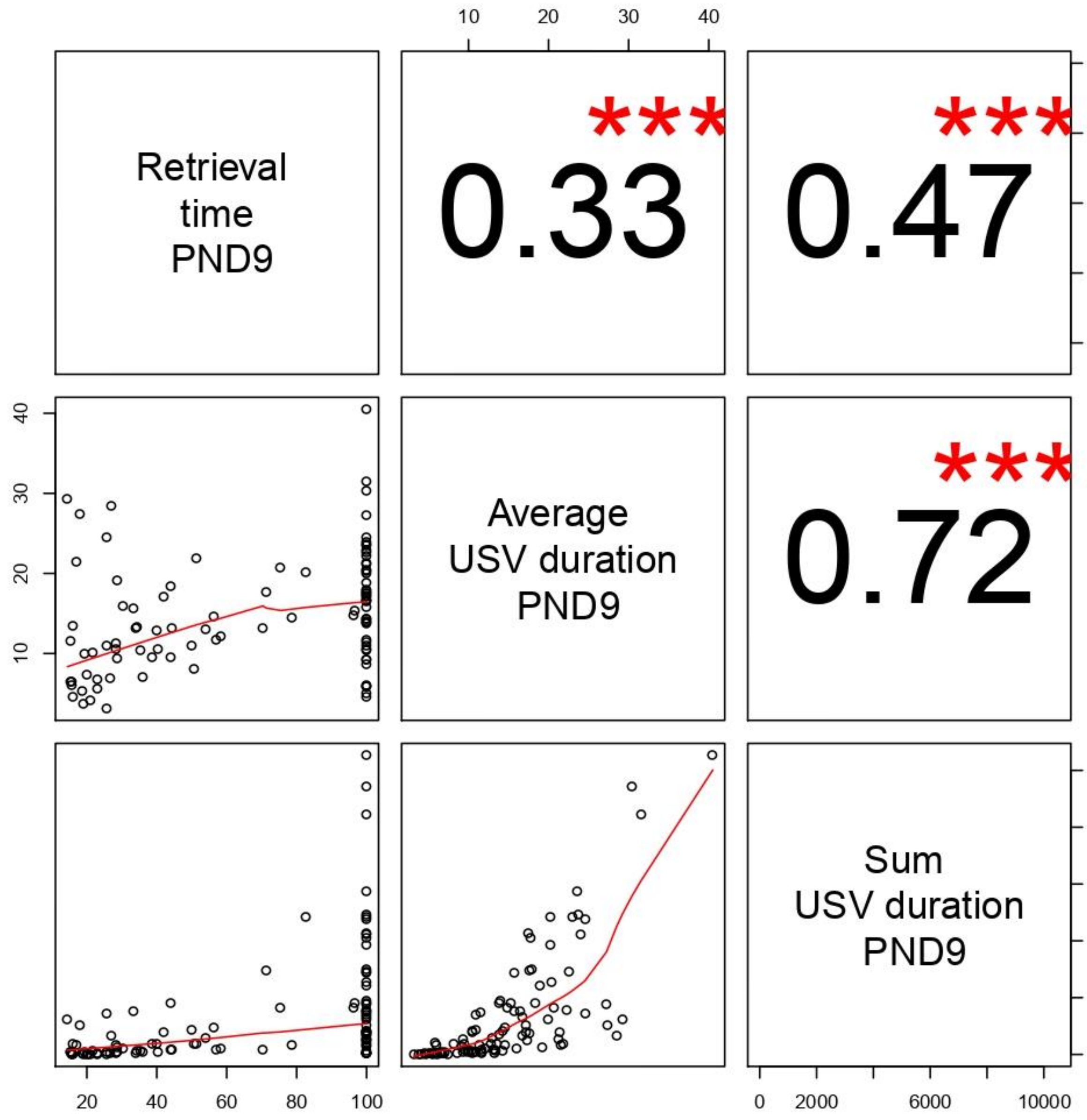
Retrieval
time
PND9
0.33
***
0.47
***
Average
USV duration
PND9
0.72
***
Sum
USV duration
PND9
10
20
30
40
100
80
60
40
20
10000
6000
2000
0
0
2000
6000
10000

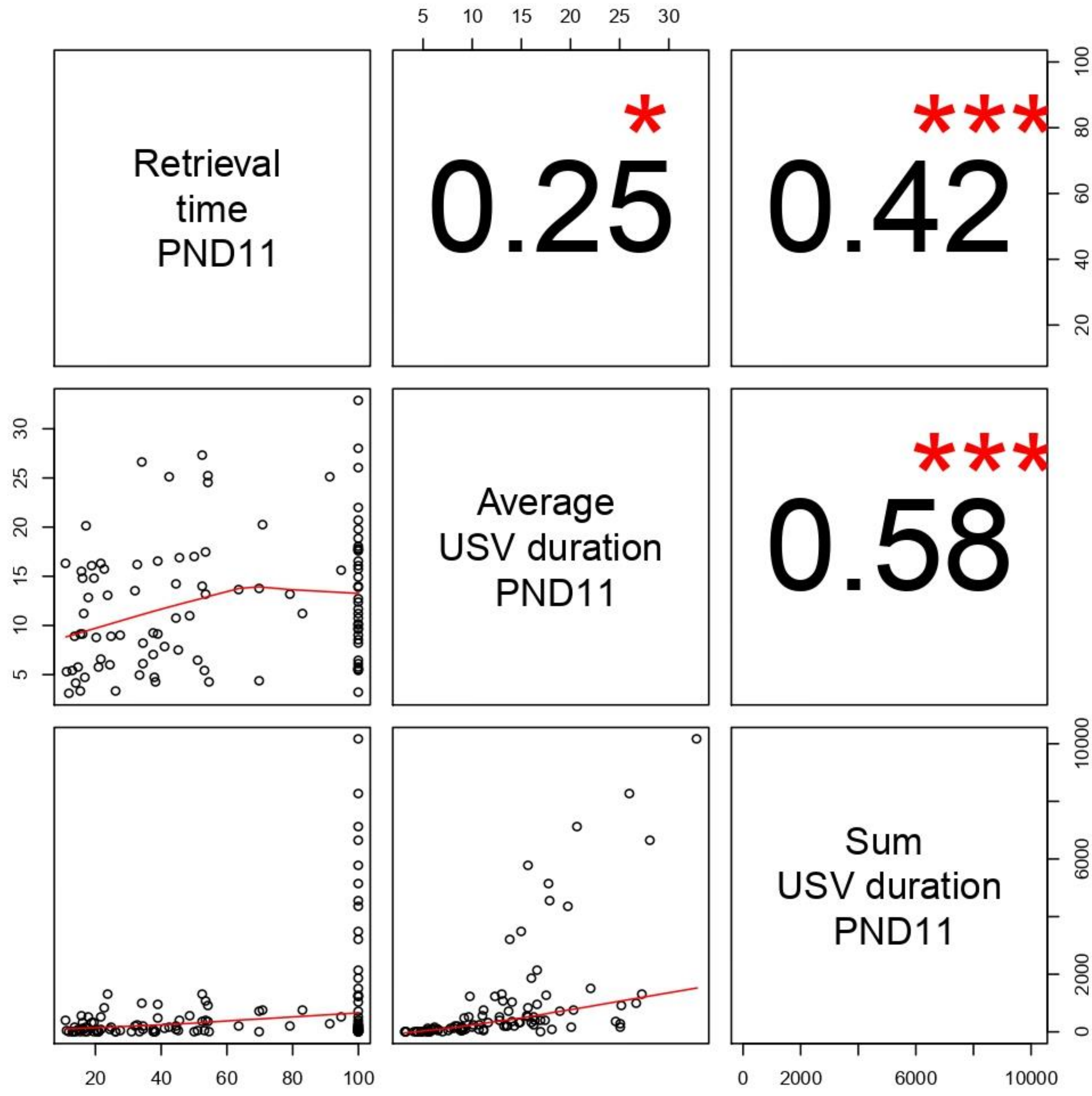

Retrieval time PND11
Average USV duration PND11
Sum USV duration PND11
0.25
*
0.42
***
0.58
***
5
10
15
20
25
30
100
80
60
40
20
10000
6000
2000
0
20
40
60
80
100
0
2000
6000
10000

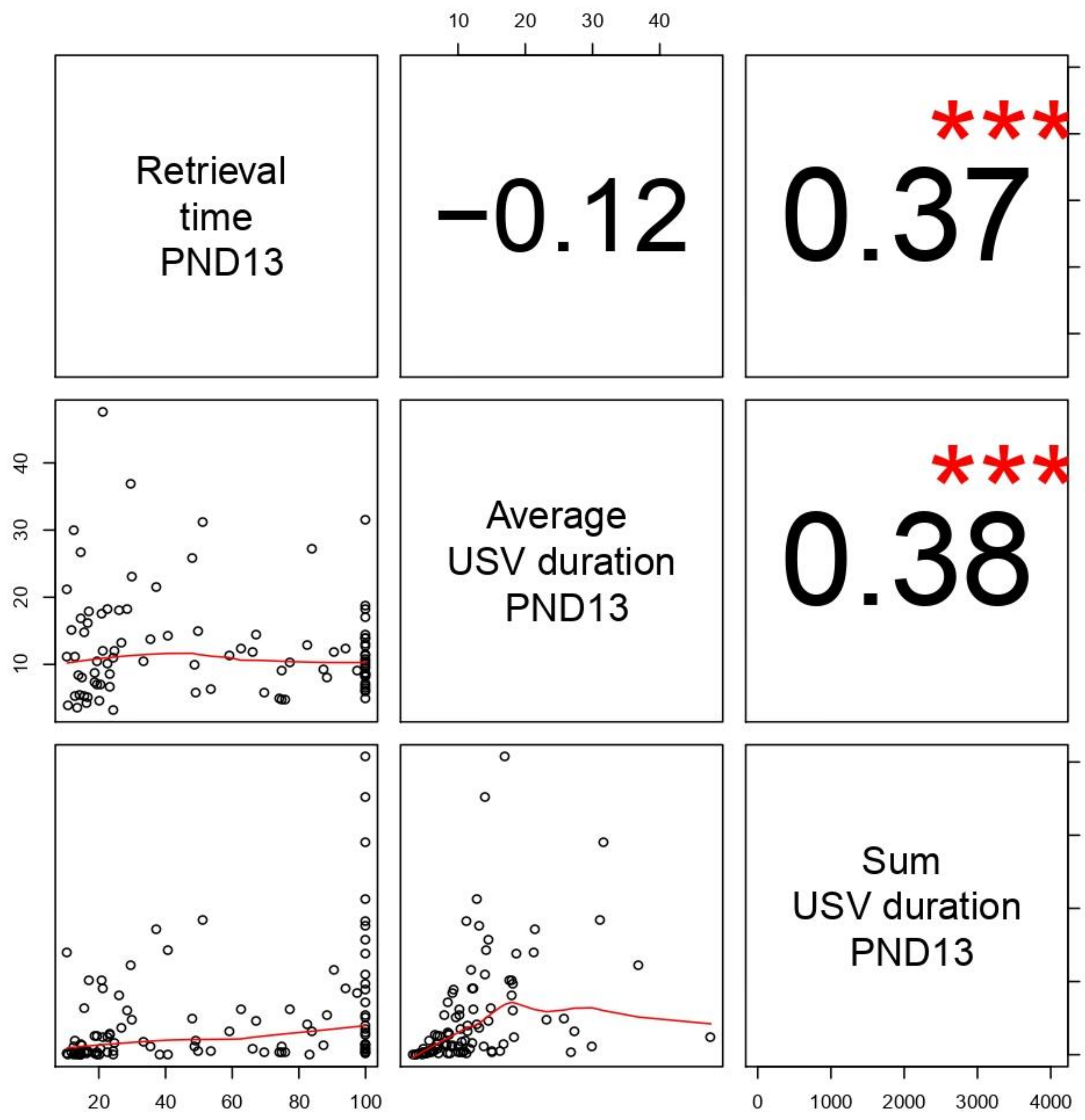
Retrieval time PND13
−0.12
0.37
***
Average USV duration PND13
0.38
***
Sum USV duration PND13
10
20
30
40
100
80
60
40
20
0
1000
2000
3000
4000
